# Universal varicella vaccination in Denmark: Modeling public health impact, age-shift, and cost-effectiveness

**DOI:** 10.1371/journal.pgph.0001743

**Published:** 2023-04-05

**Authors:** Colleen Burgess, Salome Samant, Thomas leFevre, Carsten Schade Larsen, Manjiri Pawaskar

**Affiliations:** 1 Merck & Co., Inc., Rahway, NJ, United States of America; 2 MSD Copenhagen, Copenhagen, Denmark; 3 Department of Infectious Diseases, Aarhus University Hospital, Aarhus, Denmark; University of Cape Town, SOUTH AFRICA

## Abstract

We modeled the long-term clinical and economic impact of two-dose universal varicella vaccination (UVV) strategies in Denmark using a dynamic transmission model. The cost-effectiveness of UVV was evaluated along with the impact on varicella (including age-shift) and herpes zoster burden. Six two-dose UVV strategies were compared to no vaccination, at either short (12/15 months) or medium (15/48 months) intervals. Monovalent vaccines (V-MSD or V-GSK) for the 1^st^ dose, and either monovalent or quadrivalent vaccines (MMRV-MSD or MMRV-GSK) for the 2^nd^ dose were considered. Compared to no vaccination, all two-dose UVV strategies reduced varicella cases by 94%-96%, hospitalizations by 93%-94%, and deaths by 91%-92% over 50 years; herpes zoster cases were also reduced by 9%. There was a decline in the total number of annual varicella cases in all age groups including adolescents and adults. All UVV strategies were cost-effective compared to no vaccination, with ICER values ranging from €18,228-€20,263/QALY (payer perspective) and €3,746-€5,937/QALY (societal perspective). The frontier analysis showed that a two-dose strategy with V-MSD (15 months) and MMRV-MSD (48 months) dominated all other strategies and was the most cost-effective. In conclusion, all modeled two-dose UVV strategies were projected to substantially reduce the clinical and economic burden of varicella disease in Denmark compared to the current no vaccination strategy, with declines in both varicella and zoster incidence for all age groups over a 50-year time horizon.

## Introduction

Varicella (chickenpox) is an acute and highly infectious disease caused by the varicella zoster virus. Varicella infections commonly present as a generalized pruritic vesicular rash with fever and malaise. The infections are generally mild and self-limiting, but can sometimes result in complications (e.g., bacterial infection of skin and the soft tissue, pneumonia, and encephalitis) or, rarely, death [1–3]. The risk of complication and death is greatest among the unvaccinated, the immunocompromised, and other high-risk groups (infants, pregnant women, and adults) [1–3]. Lowered immunity may cause reactivation of varicella virus later in life, presenting as herpes zoster (HZ), or shingles. HZ usually manifests as a unilateral, painful, vesicular rash in a single dermatome and increases in frequency and severity with increasing age. The most common serious complication is postherpetic neuralgia with chronic persistent pain [3].

Universal varicella vaccination (UVV) is not currently part of the Danish National Immunization Program [4]. In the absence of a UVV program, the estimated varicella disease burden in Denmark is considerable, with 63,500 cases annually, mainly among those under the age of 5 years [5]. A 2016 nationwide, database study of pediatric varicella hospitalizations in Denmark using the Danish National Patient Register found the overall annual incidence of varicella-related hospitalizations lasting at least 1 day to be 11 per 100,000 children (<18 years of age), with the highest annual incidence among children aged <2 years [6]. The total annual cost of varicella in Denmark is estimated to be €7.23 million (range €6.39-€8.07 million) in 2018 Euros, of which direct costs associated with treatment of disease account for €2.58 million (range €2.36-€2.80 million) and indirect costs, i.e., productivity loss by adult patients and caregivers, account for €4.65 million (range €4.03-€5.27 million) [5].

Varicella vaccines have been proven to be safe and effective against varicella, with UVV programs leading to significant declines in varicella morbidity and mortality [2, 3, 7–11]. UVV policies vary globally, and only about half of EU/EEA countries include it in their national immunization programs [12]. The two most common concerns related to inclusion of varicella vaccination in the national immunization programs in Europe are the possibility of an age-shift in varicella leading to more cases among older individuals at risk for more severe disease, and an increase in HZ incidence due to the impact of exogenous boosting [2, 10]. The exogenous boosting hypothesis proposes that a reduction in exposure to varicella cases in the community would lead to fewer boosting events and hence lower HZ immunity, possibly leading to a higher risk of HZ reactivation and, hence, higher HZ incidence [13, 14]. However, evidence from numerous studies, including recently published long-term data with 25 years of follow-up after UVV from the US, the first country to implement UVV, does not support age-shift [2, 10, 15–17] or the impact of exogenous boosting after UVV [2, 17–19].

The objective of the present study was to model the long-term public health impact and cost-effectiveness of universal childhood varicella vaccination strategies and assess their impact on varicella age-shift and HZ incidence in Denmark over a 50-year time horizon. These results can help inform decision-making around the introduction of a UVV program in Denmark.

## Methods

### Ethics statement

This is a dynamic transmission model that modeled for varicella-related outcomes and costs for the whole population in Denmark. No primary or secondary data was collected as part of this study. All inputs were from published literature and included only anonymized data. Our study did not involve the collection, use, or transmittal of individually identifiable data. Hence, our modeling study is out of scope for both patient IRB/EC review or patient informed consent.

### Model description

We modified a previously described age-structured, deterministic, dynamic transmission model for this analysis [20]. In brief, the model is a variation of the MSEIRV (*Maternal-Susceptible-Exposed-Infected-Recovered-Vaccinated*) structure commonly used to evaluate vaccination programs (Fig A in S1 Text) [21]. The model structure as well as the parameters have been extensively updated to reflect the most recent literature, particularly related to duration of infection, case fatality rates, exogenous boosting, and health utilities (Table B in S1 Text). In addition, this model used the latest data on vaccine performance parameters derived from 10-year clinical trial data for two different vaccine formulations [22]. The detailed vaccine parameters included in this model are described in Table D in S1 Text and their derivation from 10 years of randomized controlled trial data are described elsewhere [22]. The model was calibrated to age-stratified varicella seroprevalence and HZ incidence from a proxy country, Norway, via maximum likelihood estimates method. The observed and fitted varicella seroprevalence and HZ incidence plots are shown in Fig A in S2 Text. Model assumptions regarding exogenous boosting were updated based on real-world evidence showing the impact of contact with persons with infectious varicella on rates of HZ [19]. Using data on relative incidence of HZ following household exposure to varicella, we estimated the proportion of individuals boosted and the duration of boosting (see S1 Text for additional details) to describe the temporary immunity conferred by exogenous boosting.

Additional details are provided in the Supplement regarding the model structure (S1 Text) along with the epidemiological, health resource utilization, cost and health utility parameters used (S2 Text).

### Vaccination strategies

A total of six strategies (A-F) for UVV, each involving two doses of varicella vaccine, were compared to the no vaccination strategy over a 50-year time horizon (Table 1). Four varicella vaccines from two manufacturers were considered: monovalent Varivax (V-MSD) and quadrivalent ProQuad (MMRV-MSD), both manufactured by Merck & Co., Inc., Rahway, NJ, USA; and monovalent Varilrix (V-GSK) and quadrivalent Priorix-Tetra (MMRV-GSK), both manufactured by GSK, Belgium. Two vaccine doses were provided at either short (12 and 15 months) or medium (15 months and 48 months) intervals, aligning with the Danish National Immunization Program’s current schedule [4]. For all UVV strategies, vaccination coverage rates were set to 94% of those eligible for the first dose, and 89% of those eligible for the second dose, consistent with current measles-mumps-rubella (MMR) vaccination coverage rates [23]. Children who were 2–12 years of age at the time of UVV introduction were eligible for catch-up vaccination with two doses of monovalent vaccine, with coverage assumed to be 90% for each dose.

**Table 1:** Varicella vaccination strategies.

Strategy		Formulation		Age at vaccination (months)		Vaccination coverage		2-dose catch-up ^A^	
		1^st^ dose	2^nd^ dose	1^st^ dose	2^nd^ dose	1^st^ dose	2^nd^ dose ^B^	Formulation	Coverage for each dose ^B^
**Short interval**	A	V-MSD	V-MSD	12	15	94%	89%	V-MSD	90%
	B	V-GSK	V-GSK	12	15	94%	89%	V-GSK	90%
**Medium interval**	C	V-MSD	V-MSD	15	48	94%	89%	V-MSD	90%
	D	V-GSK	V-GSK	15	48	94%	89%	V-GSK	90%
	E	V-MSD	MMRV-MSD	15	48	94%	89%	V-MSD	90%
	F	V-GSK	MMRV-GSK	15	48	94%	89%	V-GSK	90%

MMRV, measles, mumps, rubella, and varicella combination vaccine; MMRV-GSK, Priorix-Tetra; MMRV-MSD, ProQuad; V, monovalent varicella vaccine; V-GSK, Varilrix; V-MSD: Varivax.

^A^ Catch-up vaccination: Children who were 2–12 years at time of UVV introduction were eligible for catch-up vaccination with two doses of monovalent vaccine. The first catch-up dose was given in the 1^st^ year of UVV introduction with the 2^nd^ catch-up dose given either at the recommended age of 2^nd^ routine dose or 1 year after the first catch-up dose, whichever is later.

^B^ Coverage reported for the eligible population for that dose.

### Model outcomes

Epidemiological and clinical outcomes included: the annual incidence rates of natural varicella, breakthrough varicella, and HZ; the cumulative numbers of varicella cases, outpatient visits, hospitalizations, and deaths over 50 years; and the cumulative numbers of wild (resulting from prior infection with natural varicella virus) and vaccine-related HZ cases and deaths. Age-stratified annual varicella incidence rates and the age distribution of cases were also reported to evaluate if there is a potential risk of age-shift of varicella infection to older populations.

This study also assessed the impact of UVV on exogenous boosting and HZ incidence. The current model included assumptions for exogenous boosting, modeled from a recent real-world study with 20 years of follow-up conducted in the UK (S1 Text).

Cost outcomes for all strategies were calculated from both payer (direct costs) and societal (direct and indirect costs) perspectives, along with incremental cost-effectiveness ratios (ICERs) for each UVV strategy compared to no vaccination. In addition, a frontier analysis was conducted to compare the effect and cost associated with the various UVV strategies, with comparisons made between strategies lying on the effectiveness frontier in the cost-effectiveness plane. All prices were updated to 2020 Euros. Discounting for costs and quality-adjusted life years (QALYs) followed the time-variable discount rate prescribed by the Danish Ministry of Finance of 3.5% for years 0–35, 2.5% for years 36–70, and 1.5% after 70 years [24].

In the absence of a formal cost-effectiveness threshold for Denmark, we used the per capita gross domestic product (GDP) approach recommended by the World Health Organization and also compared results to the cost-effectiveness threshold recommended by UK’s Joint Committee on Vaccination and Immunisation (JCVI; €23,964/QALY gained equivalent to £20,000/QALY gained), which is much lower than Denmark’s per capita gross domestic product (€53,552) [25, 26].

Additional scenario analyses explored outcomes at 25- and 100-year time horizons and with 3% or 5% annual discount rates (S3 Text). Additionally, the model was re-calibrated to explore outcomes in the absence of exogenous boosting. Deterministic and probabilistic sensitivity analyses were conducted on a subset of parameter values. Cost parameters were varied by ±20% and other parameters by ±5% of the baseline value. For the probabilistic analysis, 500 random parameter sets were drawn from uniform distributions.

## Results

### Impact of UVV on the clinical burden of varicella

**Fig 1:**
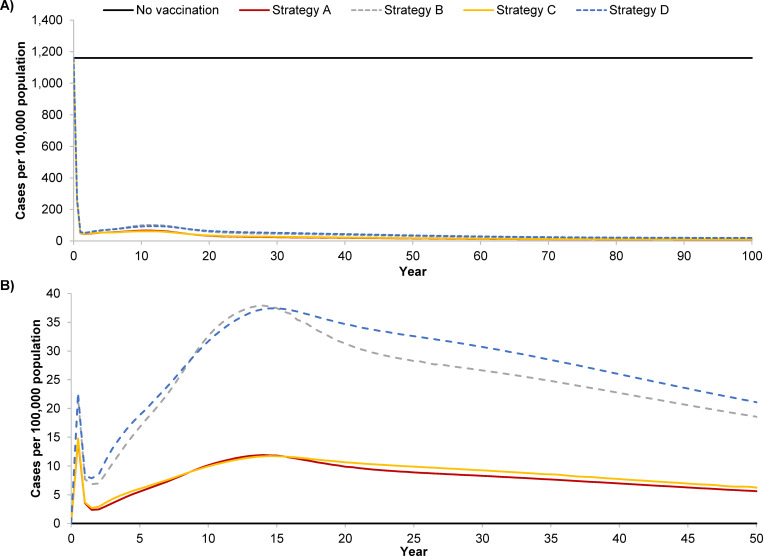
A) Total and B) breakthrough varicella incidence over time, by vaccination strategy. **Panel A:** Total varicella incidence, including natural and breakthrough cases, over 50 years after the start of universal childhood varicella vaccination. **Panel B:** Breakthrough varicella incidence over 50 years. In both panels, varicella incidence with strategies E and F were the same as for strategies C and D, respectively. **Strategy A**: V-MSD (12 months) + V-MSD (15 months); **Strategy B**: V-GSK (12 months) + V-GSK (15 months); **Strategy C**: V-MSD (15 months) + V-MSD (48 months); **Strategy D**: V-GSK (15 months) + V-GSK (48 months); **Strategy E**: V-MSD (15 months) + MMRV-MSD (48 months); **Strategy F**: V-GSK (15 months) + MMRV-GSK (48 months).

**Fig 2:**
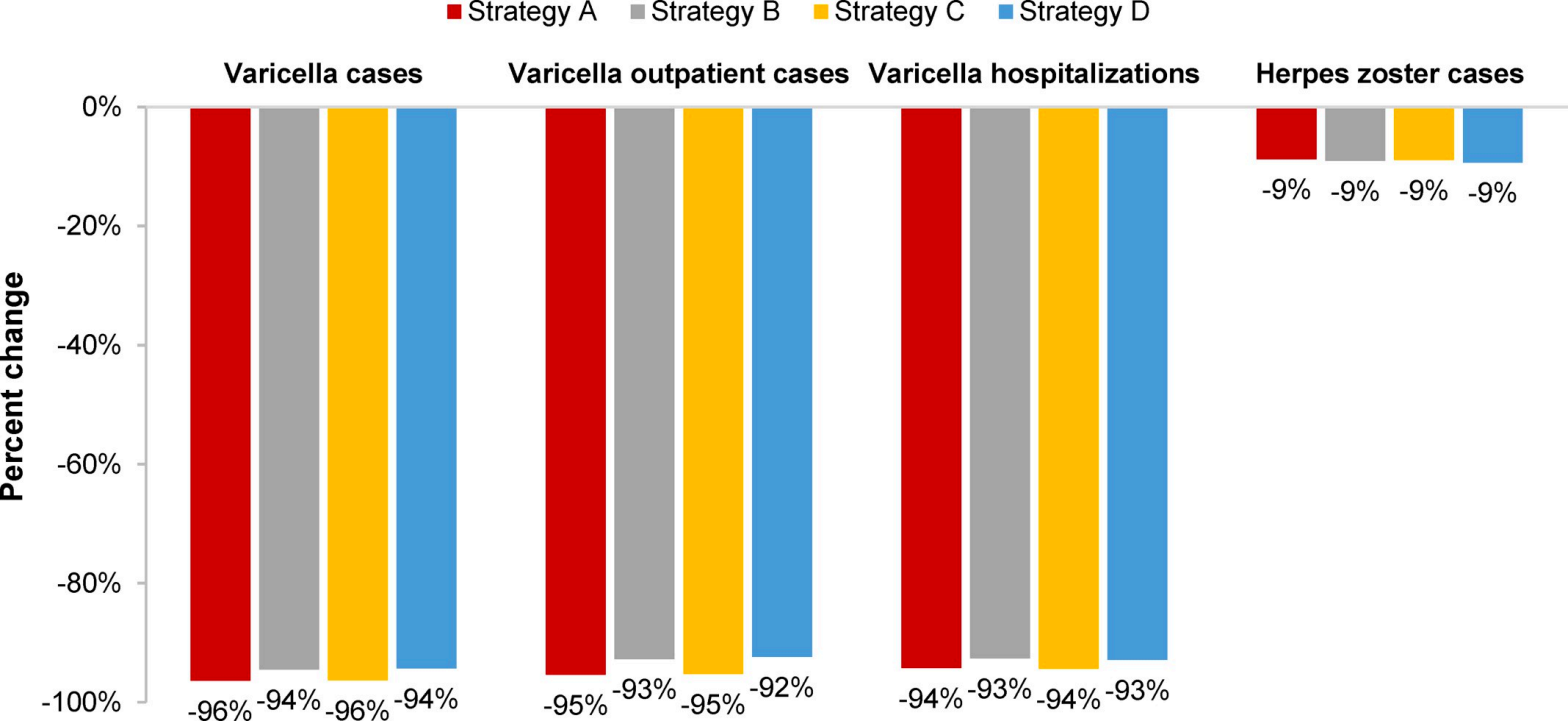
Percent change in varicella and herpes zoster health outcomes, compared to no vaccination, over 50 years, by vaccination strategy. Health outcomes with strategies E and F were the same as for strategies C and D, respectively. **Strategy A**: V-MSD (12 months) + V-MSD (15 months); **Strategy B**: V-GSK (12 months) + V-GSK (15 months); **Strategy C**: V-MSD (15 months) + V-MSD (48 months); **Strategy D**: V-GSK (15 months) + V-GSK (48 months); **Strategy E**: V-MSD (15 months) + MMRV-MSD (48 months); **Strategy F**: V-GSK (15 months) + MMRV-GSK (48 months).

All six vaccination strategies were projected to significantly reduce the annual incidence of varicella from 1,161 to 16–35 per 100,000 persons over 50 years (Fig 1A), with MSD strategies resulting in lower breakthrough varicella than GSK strategies (Fig 1B). UVV substantially decreased the burden of varicella disease, with 94%-96% of varicella cases, 93%-94% of varicella-related hospitalizations and 91%-92% of deaths averted over 50 years compared to pre-UVV (Fig 2). MSD strategies resulted in fewer total varicella cases (by 34%-36%), hospitalizations (by 21%-22%), and deaths (by 15%-16%) when compared with equivalent GSK strategies (Table 2).

**Table 2:** Varicella- and herpes zoster-related health outcomes over 50 years, by vaccination strategy ^A^.

Outcome	Vaccination strategy				
	None	A	B	C/E	D/F
**Varicella-related outcomes**					
Varicella cases					
Natural	3,404,586	99,517	112,985	99,948	113,369
Breakthrough	-	24,077	75,032	25,771	82,348
**Total varicella cases** ^**B**^	**3,404,586**	**123,594**	**188,017**	**125,719**	**195,716**
Outpatient cases	335,834	15,638	24,557	16,004	25,693
Hospitalizations	11,084	641	822	626	796
Deaths	42.75	3.35	3.97	3.23	3.80
**Herpes zoster-related outcomes**					
Herpes zoster cases					
Wild	723,945	632,662	633,247	632,789	633,468
Vaccine-type	-	28,122	25,570	27,031	23,334
** Total herpes zoster cases**	**723,945**	**660,784**	**658,818**	**659,820**	**656,802**
Herpes zoster deaths	233	231	231	231	231

^A^ Clinical outcomes with strategies E and F were the same as with strategies C and D, respectively.

^B^ Total varicella cases include those not seeking medical care.

**Strategy A**: V-MSD (12 months) + V-MSD (15 months); **Strategy B**: V-GSK (12 months) + V-GSK (15 months); **Strategy C**: V-MSD (15 months) + V-MSD (48 months); **Strategy D**: V-GSK (15 months) + V-GSK (48 months); **Strategy E**: V-MSD (15 months) + MMRV-MSD (48 months); **Strategy F**: V-GSK (15 months) + MMRV-GSK (48 months).

### Impact of UVV on varicella age-shift

**Fig 3:**
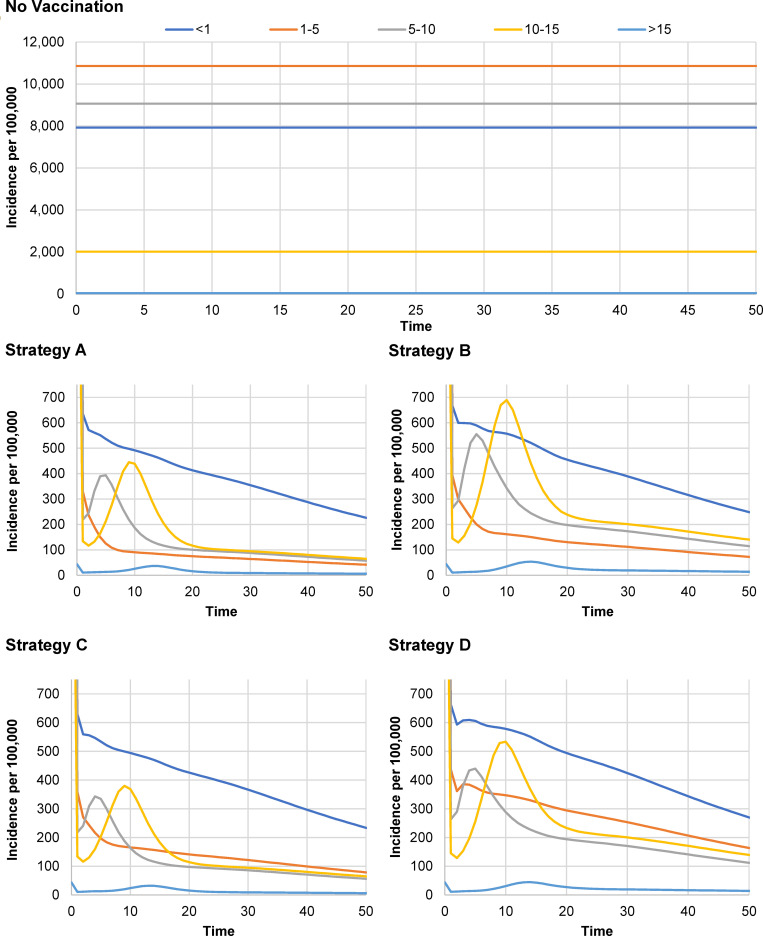
Incidence of varicella by age groups and vaccination strategy. Health outcomes with strategies E and F were the same as for strategies C and D, respectively. **Strategy A:** V-MSD (12 months) + V-MSD (15 months); **Strategy B:** V-GSK (12 months) + V-GSK (15 months); **Strategy C:** V-MSD (15 months) + V-MSD (48 months); **Strategy D:** V-GSK (15 months) + V-GSK (48 months); **Strategy E:** V-MSD (15 months) + MMRV-MSD (48 months); **Strategy F:** V-GSK (15 months) + MMRV-GSK (48 months).

In the absence of vaccination, annual varicella incidence was highest among children aged 1–5 years (10,859 per 100,000 persons) followed by incidence among children aged 5–10 years (9,065 per 100,000 persons). Immediately following the introduction of UVV, total varicella incidence declined significantly in all age groups including adolescents and adults (Fig 3). After the initial drop following the introduction of UVV, a small increase in total varicella incidence (Fig 3) was observed in children aged 5–10 years and 10–15 years in the first two decades, after which incidence again declined; the incidence in both age groups was still lower compared to no vaccination strategy. The magnitude of the increase in these age groups varied with strategies and short vs medium interval. For example, varicella incidence for MSD medium interval Strategy E for 5-10-year-olds dropped from pre-UVV values of 9,065 per 100,000 to 218 per 100,000 (263 per 100,000 for Strategy F) and then peaked at 343 per 100,000 versus 440 per 100,000 for the comparative GSK Strategy F (Fig 3). However, varicella incidence in all age groups was substantially lower for all modeled strategies compared to no vaccination, for every year following UVV introduction. Fig A in S3 Text shows annual varicella cases by age groups over time. There was significant decline in varicella cases after UVV including among children aged >10 years, even though their relative proportion was higher.

### Impact of UVV on the clinical burden of herpes zoster

All six UVV strategies projected a decline in HZ incidence of 25%-26% at 50 years post-UVV, compared to no vaccination (Table 2). The model projected a small, transient increase of less than 1% in HZ incidence, peaking about 3–4 years after UVV introduction, followed by a consistent decline beginning at about 5–10 years (Fig B in S3 Text). The cumulative number of HZ cases over 50 years was projected to decrease by approximately 9%, regardless of UVV strategy (Fig 2), from 723,945 HZ cases under no vaccination strategy to 656,802–660,784 HZ cases with UVV (Table 2).

### Cost-effectiveness of UVV

In the absence of varicella vaccination, the cumulative cost to manage varicella and HZ disease in Denmark over 50 years was projected to be €1.1 billion from the payer perspective and €1.2 billion from the societal perspective (Table 3). Compared to no vaccination, each strategy increased total costs (11.0%-12.1% from the payer perspective and 2.1%-3.2% from the societal perspective), and led to projected gains in QALYs of 6,750–6,813, to give ICER values of €18,228-€20,263/QALY from the payer perspective and €3,746-€5,937/QALY from the societal perspective. All UVV strategies were cost-effective compared to no vaccination (Table 3). In frontier analysis, Strategy E dominated all other vaccination strategies and was found to be cost-effective at a threshold of 1 x GDP per capita as well as JCVI threshold from both payer (ICER: €18,228/QALY) and societal perspectives (ICER: €3,746/QALY).

**Table 3:** Cost-effectiveness results of base case for varicella vaccination over 50 years, from payer and societal perspectives.

			Base case analysis (vs. no UVV)			Frontier analysis
Vaccination strategy	Total QALYs lost	Total cost (€)	QALYs gained ^A^	Incremental cost (€) ^A^	ICER(€/QALY) ^A^	
**Payer perspective**						
No vaccination	15,606	1,128,148,134	-	-	-	On the frontier
B	8,856	1,264,929,333	6,750	136,781,199	20,263	Strongly dominated
F	8,847	1,259,104,433	6,759	130,956,299	19,375	Strongly dominated
D	8,847	1,258,011,742	6,759	129,863,608	19,213	Strongly dominated
A	8,799	1,260,126,862	6,807	131,978,728	19,388	Strongly dominated
C	8,794	1,253,723,630	6,813	125,575,496	18,433	Strongly dominated
E	8,794	1,252,328,316	6,813	124,180,182	18,228	18,228 ^B^
**Societal perspective**						
No vaccination	15,606	1,238,054,853	-	-	-	On the frontier
B	8,856	1,278,129,988	6,750	40,075,134	5,937	Strongly dominated
F	8,847	1,272,605,457	6,759	34,550,604	5,112	Strongly dominated
D	8,847	1,271,512,888	6,759	33,458,035	4,950	Strongly dominated
A	8,799	1,271,271,278	6,807	33,216,425	4,880	Strongly dominated
C	8,794	1,264,968,757	6,813	26,913,904	3,951	Strongly dominated
E	8,794	1,263,573,368	6,813	25,518,515	3,746	3,746 ^B^

QALYs, quality-adjusted life years gained; ICER, incremental cost-effectiveness ratio.

^A^ Incremental costs, effects, and cost-effectiveness ratio relative to no vaccination.

^B^ Compared to no vaccination strategy. All other UVV strategies are strongly dominated by Strategy E since Strategy E resulted in lower costs and fewer QALYs lost compared to the other UVV strategies (A-D and F).

**Strategy A**: V-MSD (12 months) + V-MSD (15 months); **Strategy B**: V-GSK (12 months) + V-GSK (15 months); **Strategy C**: V-MSD (15 months) + V-MSD (48 months); **Strategy D**: V-GSK (15 months) + V-GSK (48 months); **Strategy E**: V-MSD (15 months) + MMRV-MSD (48 months); **Strategy F**: V-GSK (15 months) + MMRV-GSK (48 months).

### Uncertainty analyses

**Fig 4:**
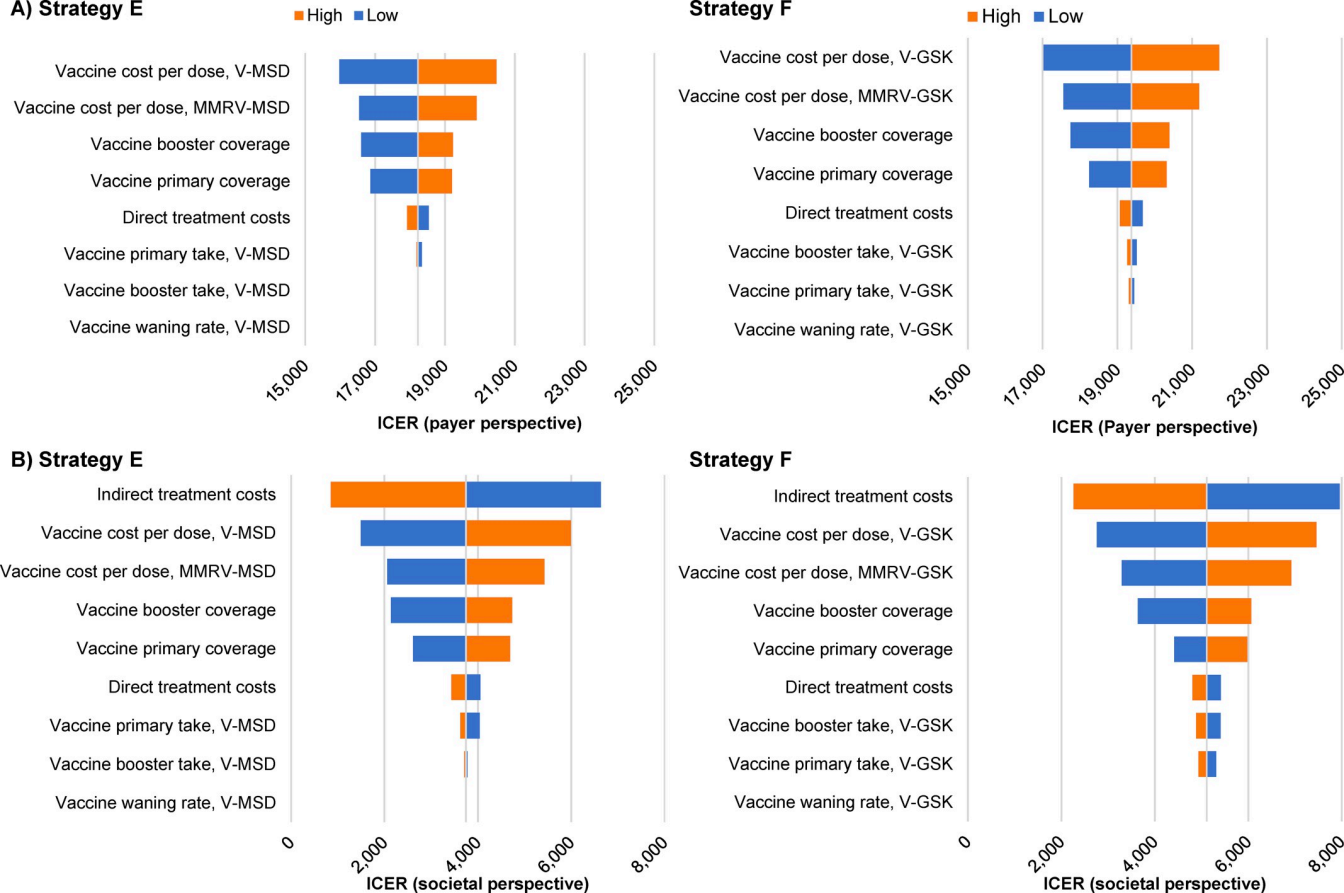
One-way sensitivity analysis on incremental cost-effectiveness ratio for vaccination Strategy E and F, from the A) payer and B) societal perspectives. Blue and orange bars show the ICER under the lower and upper parameter value assumptions, respectively. **Strategy E**: V-MSD (15 months) + MMRV-MSD (48 months). **Strategy F:** V-GSK (15 months) + MMRV-GSK (48 months).

**Fig 5:**
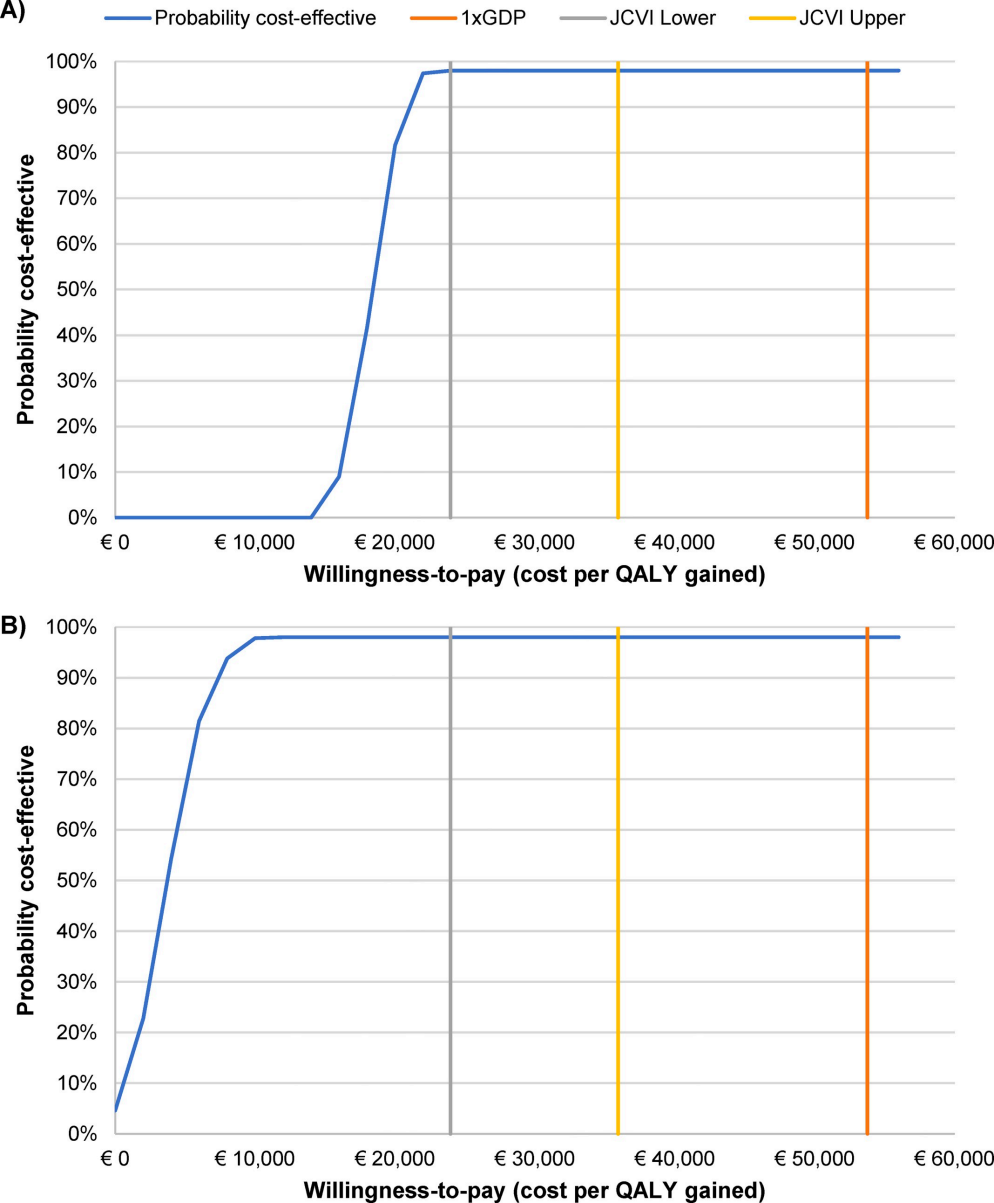
Cost-effectiveness acceptability curve for vaccination Strategy E versus no vaccination, from the A) payer and B) societal perspectives. **Strategy E**: V-MSD (15 months) + MMRV-MSD (48 months). Costs in Euros.

In the one-way deterministic sensitivity analysis, the cost-effectiveness ratios compared with no vaccination remained relatively stable to parameter variation, with values ranging between €15,975 and €20,481/QALY from the payer perspective for Strategy E (Fig 4A; and Fig C in S3 Text). The most influential parameters were the cost of monovalent and quadrivalent vaccine and primary and booster vaccination coverage rates. From the societal perspective, the ICER for Strategy E remained between €854 and €6,638/QALY gained, and the three most influential parameters were indirect treatment cost, and monovalent and quadrivalent vaccine dose costs (Fig 4B; and Fig D in S3 Text). Cost-effectiveness of UVV was robust to variations in parameters included in the deterministic sensitivity analysis for both payer and societal perspectives (Figs C and D in S3 Text), and ICERs under all evaluated parameter ranges were still well under the 1 x GDP threshold. ICER point clouds for the probabilistic sensitivity analysis also remained below the 1 x GDP threshold for all parameter variations. The cost-effectiveness acceptability curve for Strategy E is shown in Fig 5; the probabilistic sensitivity analysis point clouds for all strategies from payer and societal perspectives are shown in Figs E and F in S3 Text, respectively.

### Scenario analyses

Under the assumption of no exogenous boosting, there was no transient increase in HZ cases following introduction of varicella vaccination as observed in the base case. All strategies resulted in slightly greater reductions in HZ cases (10.4%-10.9%) and deaths (2.0%) without exogenous bosting than when exogenous boosting was included. Strategy E remained the most cost-effective vaccination strategy, with ICER values of €17,852 and €3,465/QALY gained from payer and societal perspectives, respectively (Table D in S3 Text). Similarly, even with scenarios including 3% or 5% annual discounting (Table E in S3 Text) and 25- and 100-year time horizons (Table F in S3 Text), vaccination Strategy E remained the most cost-effective strategy from both the payer and societal perspectives. ICER values at the 100-year time horizon were lower than those at 25 or 50 years, showing that vaccination strategies become even more cost-effective (payer perspective) or cost saving (societal perspective) over time (Table F in S3 Text).

## Discussion

This modeling study evaluated the cost-effectiveness of UVV in Denmark and addressed common concerns for implementing UVV in Nordic countries, namely the impact on age-shift and exogenous boosting. Our study found that all six strategies for introducing 2-dose UVV in Denmark would substantially decrease the clinical burden of varicella and were shown to be cost-effective compared to the no vaccination strategy, with Strategy E being the dominant strategy, from both payer and societal perspectives. Our study further reported no varicella age-shift to older age groups and showed a reduction in HZ incidence over 50 years which is consistent with recent literature [2, 10, 15–19].

Compared to no vaccination, all two-dose UVV strategies reduced varicella cases by 94%-96%, hospitalizations by 93%-94%, and deaths by 91%-92% over 50 years while HZ cases decreased by 9%. We found that all three strategies with MSD vaccines resulted in lower varicella incidence, breakthrough cases, and mortality when compared with equivalent strategies with GSK vaccines. This is likely due to the higher first dose permanent protection or “take” of the MSD vaccines (90.3%) compared to the GSK vaccines (61.7%) [22], and is consistent with observational studies [27–29] as well as data reported in randomized controlled trials (1^st^ dose efficacy of V-MSD: 94.4% [30]; V-GSK: 67.2% [31]).

All six short and medium interval strategies were cost-effective compared to no vaccination. The cost-effectiveness frontier analysis further provided information on which strategy was cost-effective among all strategies under consideration. However, the medium interval Strategy E (V-MSD at 15 months and MMRV-MSD at 48 months) was the only UVV strategy on the cost-effectiveness frontier and deemed to be cost-effective at a threshold of 1 x GDP per capita from both payer and societal perspectives. The medium interval strategies align with the current MMR schedule in Denmark, thus possibly giving these strategies greater weight when considering adopting UVV in Denmark.

This study assessed whether an age-shift in varicella cases would occur following the introduction of UVV, a common concern of implementing UVV in many countries [2, 10, 17]. Our model showed that varicella incidence in all age groups declined by 97%-99% for all strategies, compared with no vaccination over 50 years and did not predict any significant age-shift after UVV introduction. There was a small increase in incidence in children aged 5–15 years with all vaccination strategies during the first two decades which was still well below pre-UVV incidence. These results align with real-world studies showing significant declines in varicella incidence and varicella-related hospitalizations following UVV in all age groups, further establishing that there is no evidence of age-shift after UVV [2, 10, 15–17].

Some previously published mathematical models, including those by Brisson et al and van Hoek et al, had predicted that UVV would lead to an increase in the burden of HZ due to assumed pronounced exogenous boosting effect, and further concluded that UVV may not be a cost-effective strategy [14, 32, 33]. The effect of UVV on HZ is strongly correlated with assumptions about the magnitude and duration of exogenous boosting, both of which are subject to significant uncertainty [34]. Brisson et al and van Hoek et al both assumed magnitude of exogenous boosting effect is 100% (versus 33.45% in our model) with the duration of immunity against zoster ranging from 20 years to lifetime versus 81.3 years in our model. Recent literature has shown that the impact of UVV on exogenous boosting may not be as significant as previously thought [2, 18, 19]. For example, a study presenting 25 years of real-world data from the US (which introduced 1 dose UVV in 1995 and 2 dose UVV in 2007) did not show an increase in HZ incidence attributable to UVV and seems to indicate that rates of HZ will decline as vaccinated children age [17, 35]. The exogenous boosting assumptions used by our model were calculated from a recently published real-world study with 20 years of follow-up that examined the impact of contact with persons with infectious varicella on rates of HZ (See S1 Text for more details) [19]. In contrast with previous models, due to use of latest data, our results showed total HZ incidence was projected to decrease by about 25% by 50 years, with 9% fewer total HZ cases over the 50-year period compared to no UVV. Using current data, our model estimated all vaccination strategies to be cost-effective compared with no vaccination after accounting for the impact on exogenous boosting and HZ incidence, from both the payer and societal perspectives, considering both the JCVI and GDP thresholds. Another strength of our model compared to previous models, is the use of recently updated vaccine performance parametrization by Pillsbury et al [22], which used a new deterministic model and 10 years of follow-up to clinical trial data for both V-MSD [30] and V-GSK [31] rather than van Hoek et al’s model which only modeled V-MSD, thus allowing for the direct comparison of vaccine performance [32].

The results of our model are robust to variation in input parameters, and are consistent with recently published results for Turkey, Italy, Switzerland, UK and Norway, which showed significant reductions in the clinical and economic burden of disease after the introduction of UVV [20, 36–39]. This is also consistent with a cost analysis done by the Danish Chamber of Commerce, which found annual cost savings of 169 million Danish Krone (€22.7 million) with a two-dose varicella vaccination strategy from the societal perspective [40]. In addition to the cost-effectiveness of vaccination strategies, several factors need to be considered when implementing UVV [41]. These include the ability to maintain high vaccination coverage (>80%), the flexibility of the pediatric vaccination schedule to accommodate an additional vaccination visit (if required), fiscal vaccination budget, as well as the programmatic goals of the country [3].

### Limitations

Modeling studies incorporate various assumptions about which there is uncertainty. We used proxy data from Norway and Sweden when recent Danish data were unavailable, though the similarity between these Nordic countries suggests they can be appropriate substitutes. Since the list prices for MMRV formulations in Denmark were not available, we estimated list prices from the International Reference Pricing basket using an approach recommended by the Danish Ministry of Health [42]. List prices tend to be higher than tender prices; hence ours is a more conservative estimate of cost-effectiveness. It was assumed that, when the second vaccination occurred as a combination MMRV vaccine, the effectiveness of the quadrivalent vaccines was the same as that of a monovalent varicella vaccine dose. We used the latest real-world evidence to estimate the impact of exogenous boosting, however different data or exogenous boosting assumptions might lead to different HZ outcomes. Additionally, our model did not account for changes in the age distribution of the population over time. This may impact the results especially since Denmark, like other European countries, has an aging population. Finally, the model assumed zero productivity loss for both varicella and HZ in adults 65 years and older, possibly underestimating true societal cost savings of UVV.

## Conclusions

All modeled two-dose strategies of universal childhood varicella vaccination were projected to substantially reduce the clinical burden of varicella and were cost-effective compared to no vaccination in Denmark. Our model also showed no significant age-shift, and varicella incidence was substantially reduced across all age groups including adolescents and adults compared to no vaccination. Although there was a small increase in HZ incidence within the first few years of UVV introduction, overall HZ incidence decreased over 50-year time horizon. While all vaccination strategies were cost-effective compared to no vaccination, the medium interval strategy, V-MSD at 15 months and MMRV-MSD at 48 months, was the most cost-effective from payer and societal perspectives. These results provide evidence to support the introduction of universal childhood two-dose varicella vaccination in Denmark.

## Supporting information

S1 Text Model description. (DOCX)

S2 Text Model inputs specific for Denmark. (DOCX)

S3 Text Model results. (DOCX)

## References

[1] European Centre for Disease Prevention and Control. Varicella vaccination in the European Union Stockholm: ECDC; 2015. Available from: https://www.ecdc.europa.eu/sites/default/files/media/en/publications/Publications/Varicella-Guidance-2015.pdf.

[2] Wutzler P, Bonanni P, Burgess M, Gershon A, Sáfadi MA, Casabona G. Varicella vaccination—the global experience. Expert Rev Vaccines. 2017;16:(8). doi: 10.1080/14760584.2017.1343669 28644696 PMC5739310

[3] Varicella and herpes zoster vaccines: WHO position paper, June 2014. Wkly Epidemiol Rec. 2014;89:(25). 24983077

[4] European Centre for Disease Prevention and Control. Vaccine Scheduler 2020 updated December 16, 2020. Available from: https://vaccine-schedule.ecdc.europa.eu/Scheduler/ByCountry?SelectedCountryId=58&IncludeChildAgeGroup=true&IncludeChildAgeGroup=false&IncludeAdultAgeGroup=true&IncludeAdultAgeGroup=false.

[5] Pawaskar M, Méroc E, Samant S, Flem E, Bencina G, Riera-Montes M, et al. Economic burden of varicella in Europe in the absence of universal varicella vaccination. BMC public health. 2021;21:(1). doi: 10.1186/s12889-021-12343-x PMC8690977 34930179

[6] Helmuth IG, Poulsen A, Molbak K. A national register-based study of paediatric varicella hospitalizations in Denmark 2010–2016. Epidemiology and infection. 2017. doi: 10.1017/S0950268817001777 28803571 PMC9203448

[7] Marin M, Marti M, Kambhampati A, Jeram SM, Seward JF. Global Varicella Vaccine Effectiveness: A Meta-analysis. Pediatrics. 2016;137:(3). doi: 10.1542/peds.2015-3741 26908671

[8] Zhou F, Harpaz R, Jumaan AO, Winston CA, Shefer A. Impact of varicella vaccination on health care utilization. JAMA. 2005;294:(7). doi: 10.1001/jama.294.7.797 16106004

[9] Centers for Disease Control and Prevention. Chickenpox Vaccine Saves Lives and Prevents Serious Illness Infographic. Available from: https://www.cdc.gov/chickenpox/vaccine-infographic.html.

[10] Varela FH, Pinto LA, Scotta MC. Global impact of varicella vaccination programs. Hum Vaccin Immunother. 2019;15:(3). doi: 10.1080/21645515.2018.1546525 30427766 PMC6605725

[11] Marin M, Lopez AS, Melgar M, Dooling K, Curns AT, Leung J. Decline in Severe Varicella Disease During the United States Varicella Vaccination Program: Hospitalizations and Deaths, 1990–2019. J Infect Dis. 2022;226:(Suppl 4). doi: 10.1093/infdis/jiac242 36265852 PMC10406340

[12] European Centre for Disease Prevention and Control. Vaccine Scheduler. Available from: https://vaccine-schedule.ecdc.europa.eu/Scheduler/ByDisease?SelectedDiseaseId=11&SelectedCountryIdByDisease=-1.

[13] Hope-Simpson RE. The nature of herpes zoster: A long-term study and a new hypothesis. Proceedings of the Royal Society of Medicine. 1965;58 14267505 10.1177/003591576505800106 PMC1898279

[14] Brisson M, Edmunds WJ, Gay NJ, Law B, De Serres G. Modelling the impact of immunization on the epidemiology of varicella zoster virus. Epidemiology and infection. 2000;125:(3). doi: 10.1017/s0950268800004714 11218215 PMC2869648

[15] Baxter R, Tran TN, Ray P, Lewis E, Fireman B, Black S, et al. Impact of vaccination on the epidemiology of varicella: 1995–2009. Pediatrics. 2014;134:(1). doi: 10.1542/peds.2013-4251 24913796

[16] Marin M, Leung J, Anderson TC, Lopez AS. Monitoring Varicella Vaccine Impact on Varicella Incidence in the United States: Surveillance Challenges and Changing Epidemiology, 1995–2019. J Infect Dis. 2022;226:(Suppl 4). doi: 10.1093/infdis/jiac221 36265855

[17] Marin M, Seward JF, Gershon AA. 25 Years of Varicella Vaccination in the United States. J Infect Dis. 2022;226:(Suppl 4). doi: 10.1093/infdis/jiac251 PMC10310989 36265845

[18] Harpaz R. Do varicella vaccination programs change the epidemiology of herpes zoster? A comprehensive review, with focus on the United States. Expert Rev Vaccines. 2019;18:(8). doi: 10.1080/14760584.2019.1646129 31318605

[19] Forbes H, Douglas I, Finn A, Breuer J, Bhaskaran K, Smeeth L, et al. Risk of herpes zoster after exposure to varicella to explore the exogenous boosting hypothesis: self controlled case series study using UK electronic healthcare data. BMJ. 2020;36810.1136/bmj.l6987. doi: 10.1136/bmj.l6987 31969318 PMC7190015

[20] Wolfson LJ, Daniels VJ, Pillsbury M, Kurugöl Z, Yardimci C, Kyle J, et al. Cost-effectiveness analysis of universal varicella vaccination in Turkey using a dynamic transmission model. PLoS One. 2019;14:(8). doi: 10.1371/journal.pone.0220921 31408505 PMC6692038

[21] Schuette MC, Hethcote HW. Modeling the effects of varicella vaccination programs on the incidence of chickenpox and shingles. Bulletin of mathematical biology. 1999;61:(6). doi: 10.1006/bulm.1999.0126 17879870

[22] Pillsbury M, Carias C, Samant S, Greenberg D, Pawaskar M. Comparison of performance of varicella vaccines via infectious disease modeling. Vaccine. 2022. doi: 10.1016/j.vaccine.2022.05.003 35660037

[23] Sundhedsstryelsen. Bornevaccinations-programmet, 2018. Available from: https://www.sst.dk/da/nyheder/2019/~/media/02CBB557937E4218AE5F742CA642FA9B.ashx.

[24] Finansministeriet. Dokumentationsnotat–den samfundsøkonomiske diskonteringsrente 2021. Available from: https://fm.dk/media/18371/dokumentationsnotat-for-den-samfundsoekonomiske-diskonteringsrente_7-januar-2021.pdf

[25] The World Bank. GDP per capita (current US$)—Denmark, 1996–2020. Available from: https://data.worldbank.org/indicator/NY.GDP.PCAP.CD?locations=DK.

[26] Internal Revenue Service. Yearly Average Exchange Rates for Converting Foreign Currencies into US Dollars 2021. Available from: https://www.irs.gov/individuals/international-taxpayers/yearly-average-currency-exchange-rates.

[27] Spackova M, Wiese-Posselt M, Dehnert M, Matysiak-Klose D, Heininger U, Siedler A. Comparative varicella vaccine effectiveness during outbreaks in day-care centres. Vaccine. 2010;28:(3). doi: 10.1016/j.vaccine.2009.10.086 19874924

[28] Baxter R, Ray P, Tran TN, Black S, Shinefield HR, Coplan PM, et al. Long-term effectiveness of varicella vaccine: a 14-Year, prospective cohort study. Pediatrics. 2013;131:(5). doi: 10.1542/peds.2012-3303 23545380

[29] Pawaskar M, Siddiqui MK, Takyar J, Sharma A, Fergie J. Relative efficacy of varicella vaccines: Network meta-analysis of randomized controlled trials. Curr Med Res Opin. 2022. doi: 10.1080/03007995.2022.2091334 35713564

[30] Kuter B, Matthews H, Shinefield H, Black S, Dennehy P, Watson B, et al. Ten year follow-up of healthy children who received one or two injections of varicella vaccine. The Pediatric infectious disease journal. 2004;23:(2). doi: 10.1097/01.inf.0000109287.97518.67 14872179

[31] Povey M, Henry O, Riise Bergsaker MA, Chlibek R, Esposito S, Flodmark CE, et al. Protection against varicella with two doses of combined measles-mumps-rubella-varicella vaccine or one dose of monovalent varicella vaccine: 10-year follow-up of a phase 3 multicentre, observer-blind, randomised, controlled trial. Lancet Infect Dis. 2019;19:(3). doi: 10.1016/S1473-3099(18)30716-3 30765242

[32] van Hoek AJ, Melegaro A, Zagheni E, Edmunds WJ, Gay N. Modelling the impact of a combined varicella and zoster vaccination programme on the epidemiology of varicella zoster virus in England. Vaccine. 2011;29:(13). doi: 10.1016/j.vaccine.2011.01.037 21277405

[33] Brisson M, Gay NJ, Edmunds WJ, Andrews NJ. Exposure to varicella boosts immunity to herpes-zoster: implications for mass vaccination against chickenpox. Vaccine. 2002;20:(19–20). doi: 10.1016/s0264-410x(02)00180-9 12057605

[34] Poletti P, Melegaro A, Ajelli M, Del Fava E, Guzzetta G, Faustini L, et al. Perspectives on the impact of varicella immunization on herpes zoster. A model-based evaluation from three European countries. PLoS One. 2013;8:(4). doi: 10.1371/journal.pone.0060732 23613740 PMC3629254

[35] Leung J, Dooling K, Marin M, Anderson TC, Harpaz R. The Impact of Universal Varicella Vaccination on Herpes Zoster Incidence in the United States: Comparison of Birth Cohorts Preceding and Following Varicella Vaccination Program Launch. J Infect Dis. 2022;226:(Suppl 4). doi: 10.1093/infdis/jiac255 36265856

[36] Azzari C, Baldo V, Giuffrida S, Gani R, O’Brien E, Alimenti C, et al. The Cost-Effectiveness of Universal Varicella Vaccination in Italy: A Model-Based Assessment of Vaccination Strategies. ClinicoEconomics and outcomes research: CEOR. 2020;1210.2147/ceor.s229685. doi: 10.2147/CEOR.S229685 32606844 PMC7294569

[37] Heininger U, Pillsbury M, Samant S, Lienert F, Guggisberg P, Gani R, et al. Health Impact and Cost-effectiveness Assessment for the Introduction of Universal Varicella Vaccination in Switzerland. The Pediatric infectious disease journal. 2021;40:(6). doi: 10.1097/INF.0000000000003136 33872276

[38] Pawaskar M, Burgess C, Pillsbury M, Wisløff T, Flem E. Clinical and economic impact of universal varicella vaccination in Norway: A modeling study. PLoS One. 2021;16:(7). doi: 10.1371/journal.pone.0254080 34237090 PMC8266049

[39] Sharomi O, Xausa I, Nachbar R, Pillsbury M, Matthews I, Petigara T, et al. Modeling the Impact of Exogenous Boosting and Universal Varicella Vaccination on the Clinical and Economic Burden of Varicella and Herpes Zoster in a Dynamic Population for England and Wales. Vaccines. 2022;10:(9). 10.3390/vaccines10091416 PMC9501498 36146493

[40] Erhverv D. Samfundsøkonomisk potentiale ved vaccination 2020. Available from: https://www.danskerhverv.dk/siteassets/mediafolder/dokumenter/01-analyser/analysenotater-2020/samfundsokonomisk-potentiale-ved-vaccination.pdf.

[41] Bertram MY, Lauer JA, De Joncheere K, Edejer T, Hutubessy R, Kieny MP, et al. Cost-effectiveness thresholds: pros and cons. Bulletin of the World Health Organization. 2016;94:(12). doi: 10.2471/BLT.15.164418 27994285 PMC5153921

[42] Agreement of price reductions and a cap on the prices of hospital-only medicinal products for the period April 1, 2019 –March 31, 2023. Available from: https://www.lif.dk/wp-content/uploads/2020/10/Price-cap-agreement-on-hospital-medicin-2019-2023.pdf.

