# Correction: Universal varicella vaccination in Denmark: Modeling public health impact, age-shift, and cost-effectiveness

**DOI:** 10.1371/journal.pgph.0002407

**Published:** 2023-09-14

**Authors:** Colleen Burgess, Salome Samant, Thomas leFevre, Carsten Schade Larsen, Manjiri Pawaskar

In the Funding section, the role of the funder is incorrect. The correct Funding statement is: This study was funded by Merck Sharp & Dohme LLC, a subsidiary of Merck & Co., Inc., Rahway, NJ, USA. The funder provided support in the form of salaries or consulting fees for CB, SS, TL, CSL, and MP, and had input in the study design, data collection and analysis, decision to publish, and preparation of the manuscript.

The authors requested this update prior to the article’s publication, but due to an internal error the updated statement was not incorporated into the article before its publication date. The publisher apologizes for the error.
