# Supplementary material for: Universal varicella vaccination in Denmark: Modeling public health impact, age-shift, and cost-effectiveness
Source: PLOS Glob Public Health. 2023 Apr 5;3(4):e0001743. doi: 10.1371/journal.pgph.0001743 (PMC10075481; doi:10.1371/journal.pgph.0001743)
Supplement: S1 Text — (DOCX) [file pgph.0001743.s001.docx]

# S1 Text: Model description

- [Compartmental model structure](#_Compartmental_model_structure)
  - Fig A. Model diagram
  - Table A. Definition of model variables
  - Table B. Definition of model demographic and epidemiological parameters

- [Force of infection](#_Force_of_infection)
  - Table C. Mapping between data and model age groups
- [Vaccination](#_Vaccination)
  - Table D. Vaccine properties
- [Estimation of boosting proportion and duration of boosting](#_Estimation_of_boosting)
  - Table E. Incidence ratio of herpes zoster following household exposure to a child with varicella
  - Fig B. Least-square fit of the incidence ratio
- [Ordinary differential equations](#_Ordinary_differential_equations)
- [References](#_References)

## Compartmental model structure

A deterministic, age-structured, continuous-time, non-linear dynamic transmission model in the form of differential equations was constructed and used to quantify and assess the potential public health impact of a varicella immunization program in the Danish population. The aging compartments in the model were structured to capture the demographic, epidemiological, behavioral, and economic data available for Denmark and vaccination dose schedules of interest.

The model itself was a variation of an MSEIRV (Maternal-Susceptible-Exposed-Infected-Recovered-Vaccinated) model, a structure commonly used to evaluate the impact of vaccination programs (1). It was extended to include health states that represent the reactivation of latent varicella zoster virus leading to HZ. Furthermore, the set of health states for tracking vaccinated persons over time were replicated to differentiate between those who received their first or second vaccination dose in the UVV program. The set of health states and corresponding transitions were similar to those used in previous cost-effectiveness analyses of UVV (2, 3, 4, 5, 6), but with changes that use the *Failure-Take-Waning-Degree* structure of vaccine effectiveness (7), which better aligns with the long-term data from randomized control trials (10 year follow-up) for V-MSD (8) and V-GSK (9). A model flowchart is given in Fig A. Model variables are defined in Table A. Epidemiological parameters are given in Table B.

Fig A. Model diagram

Blue lines represent vaccination with UVV first dose, purple lines represent vaccination with second dose, red lines represent varicella natural history and black lines represent all other transitions in the figure. Subscripts 0,1,2 refer to the number of varicella vaccination doses received. See Table A for definitions of model variables.

Newborns entered the model with passive immunity against varicella conferred by maternal antibodies $\left( m \right)$ and later became susceptible to natural varicella $\left( s \right)$ following waning of such immunity. Unvaccinated children developed latent varicella, i.e., they became exposed $(e)$, following sufficient contact with infectious individuals (including infectious cases of herpes zoster). Fourteen days after such exposure, exposed individuals became infectious with varicella $\left( i \right)$ and developed varicella disease. Individuals could either recover from varicella infection with high immunity against zoster $\left( r \right)$ or die from varicella $\left( ndv \right)$. Over time, this acquired zoster immunity in individuals from varicella would wane $\left( w \right)$. Exposure to varicella could lead back to increased immunity (exogenous boosting effect).

Children were vaccinated against varicella according to schedules detailed in the main manuscript. A varicella vaccine dose may either fail to induce an immune response $\left( s \right)$, succeed in inducing temporary immunity $\left( vq \right)$, or succeed in inducing long-lasting immunity $\left( v \right)$. In the case of vaccine failure, children remained susceptible $\left( s \right)$ to varicella infections with outcomes that are as severe as natural varicella. However, it was not possible to determine whether a vaccinated individual developing varicella disease had a primary vaccine failure or breakthrough varicella (due to waning).

The interval between the first and second dose for either routine or catch-up vaccination may be interrupted by progression to an infectious stage $\left( i \right)$ of natural varicella following exposure $\left( e \right)$. In such cases of breakthrough infection after single dose of vaccine, the child would not be eligible for the 2^nd^ dose.

Immunity gained from a successful first dose was either temporary (${vq}_{1}$) or long-lasting ($v_{1}$). Temporary immunity waned at a rate based on vaccine characteristics, and after waning, vaccinees were at risk of acquiring breakthrough varicella. If a person with waned vaccine immunity was exposed to varicella $\left( evb_{1} \right)$, they would progress to infectious varicella after breakthrough $\left( ivb \right)$. Breakthrough infectious individuals could either recover with high immunity against HZ $\left( rvb \right)$ or die from varicella $\left( ndv \right)$. Recovered individuals, again, could lose acquired immunity to zoster $\left( wvb \right)$.

For children who had received their first varicella vaccine dose in a two-dose vaccine schedule, the second dose was administered at an age determined by the vaccination strategy (see Table 1 in the main manuscript). If the second dose was successful, immunity gained was either temporary (${vq}_{2}$) or long-lasting ($v_{2}$). The rest of the dynamics followed the same trajectory as that after the first successful dose.

As immunity against HZ waned, persons previously infected with varicella zoster virus (natural infection) could undergo HZ reactivation $(z$, $zwild$). Individuals who had been previously and successfully vaccinated (against varicella) could undergo HZ reactivation as well $\left( zvacc \right)$. HZ reactivation resulted in either recovery with life-long immunity $\left( rz \right)$ or death $\left( ndz \right)$.

Throughout their lifetime, individuals could benefit from exogenous boosting. This phenomenon occurred when individuals with waned immunity against zoster were in close physical proximity to varicella-infectious individuals. Exposure to a circulating virus boosted the immune system, thus extending protection and reducing the risk of HZ reactivation (10). A decrease in exogenous boosting due to UVV could potentially cause an increase in the incidence of HZ or shift the incidence of natural varicella to older age groups who are more likely than younger individuals to experience severe illness leading to hospitalization and higher costs (11). The clinical importance and magnitude of exogenous boosting are not well-characterized, and evidence for the effect of UVV on exogenous boosting and the incidence of HZ is inconsistent (12, 13). Some prior modeling studies that evaluated UVV impact on HZ incidence with reduced or no exogenous boosting found that UVV implementation resulted in incremental cost-effectiveness ratios (ICERs) that exceeded accepted thresholds (14). This model was one in which exogenous boosting provides temporary immunity, with parameters derived from recent real-world evidence on the impact of contact with persons with infectious varicella on rates of HZ (15).

All compartments were stratified by age and the age cuts, in years, were:

$\left\{ \begin{aligned} \frac{1}{12},\frac{6}{12},\frac{11}{12},1,\frac{13}{12},\frac{14}{12},\frac{15}{12},\frac{16}{12},\frac{17}{12},\frac{18}{12},\frac{19}{12},\frac{20}{12},\frac{21}{12},\frac{22}{12},\frac{23}{12},2, 3,4, 5,6, 7, 8, 9, 10,11, 12, \\ \\ 13, 14, 15, 16, 17, 18, 19, 20, 25, 30, 35, 40, 45, 50, 55, 60, 65, 70, 75, 80, 85, 90 \end{aligned} \right\}$

There were 49 age groups in this model, indicated by the subscript $j$. For vaccinated compartments, the number of doses already administered was indicated by the subscript $l=\left\{ 1,2 \right\}.$

Table A. Definition of model variables

| **Variable** | **Description (variables represent proportions)** |
| --- | --- |
| **Unvaccinated compartments** | |
| $m_{j}$ | Passively immune |
| $s_{j}$ | Susceptible to varicella infection |
| $e_{j}$ | Latent varicella |
| $i_{j}$ | Infectious varicella |
| $r_{j}$ | Recovered from varicella with high herpes zoster immunity |
| $w_{j}$ | Low herpes zoster immunity due to waning effects |
| **Varicella vaccination** | |
| $v_{j,l}$ | Long-lasting immunity following $l-$dose vaccination |
| ${vq}_{j,l}$ | Temporary immunity following $l-$dose vaccination |
| ${vs}_{j,l}$ | Susceptible to varicella following $l-$dose vaccine waning |
| $s_{j,l}$ | Susceptible to varicella following $l-$dose vaccine failure |
| $e_{j,l}$ | Latent varicella following $l-$dose vaccine failure |
| ${rvv}_{j,l}$ | High herpes zoster immunity following $l-$dose vaccination |
| ${wvv}_{j,l}$ | Low herpes zoster immunity following $l-$dose vaccine waning |
| **Breakthrough varicella** | |
| ${evb}_{j,l}$ | Latent varicella following $l-$dose vaccine waning |
| ${ivb}_{j}$ | Infectious varicella |
| ${rvb}_{j}$ | Recovered from varicella with high herpes zoster immunity |
| ${wvb}_{j}$ | Low herpes zoster immunity due to waning |
| **Herpes zoster reactivation** | |
| $z_{j}$ | Infectious with wild type herpes zoster |
| ${zvacc}_{j}$ | Infectious with vaccine-type herpes zoster post varicella vaccination |
| ${zwild}_{j}$ | Infectious with wild type herpes zoster post breakthrough varicella |
| ${rz}_{j}$ | Recovered from herpes zoster with high herpes zoster immunity |
| **Death** | |
| ${ndv}_{j}$ | Death from varicella |
| ${ndz}_{j}$ | Death from herpes zoster |

Subscript $j$ indicates the age group.

Table B. Definition of model demographic and epidemiological parameters

| **Parameter** | **Description** | | | | **Value** | | **Source** |
| --- | --- | --- | --- | --- | --- | --- | --- |
| **Demographic rates (per year) ^A^** | | | | | | | |
| $f_{j}$ | Fertility rate | | | | Varies | | (16) |
| $\mu_{j}$ | Force of mortality ^B^ | | | | Varies | | (16) |
| -- | Sex ratio | | | | Varies | | (16) |
| $d_{j}$ | Maturation rate | | | | Varies | | Computed |
| $\beta_{j_{1},j_{2}}$ | Contact mixing rate between age groups $j_{1}$ and $j_{2}$ | | | | Varies | | (17) |
| ${rr}_{j}$ | Age-specific relative risk of varicella transmission per contact | | | | 0-3 years: 5.24483  4-19 years: 6.50375  ≥20 years: 3.03473 x 10^-7^ | | Calibrated |
| $\Lambda$ | Birth rate | | | | Varies | | Computed |
| **Natural varicella infection** | | | | | | | |
| $\frac{1}{\omega^{m}}$ | Average waning period of passive (maternally-induced) immunity | | | | 7.3 months ^C^ | | Calibrated |
| $\frac{1}{\epsilon^{n}}$ | Average latent period of natural varicella | | | | 14 days | | (18, 19) |
| $\frac{1}{\gamma^{n}}$ | Average infectious period of natural varicella | | | | 7 days | | (19, 20, 21) |
| $\frac{1}{\delta^{n}}$ | Average waning period of zoster immunity after natural varicella | | | | 81.3 years ^C^ | | (15)  [See section below](#_Estimation_of_boosting) ^D^ |
| **Breakthrough varicella infection** | | | | | | | |
| $\rho^{v}$ | Relative infectivity of breakthrough varicella | | | | 50% | | (18, 22) |
| $\frac{1}{\epsilon^{vb}}$ | Average latent period of breakthrough varicella | | | | 14 days (Assumed to be same as natural varicella) | | (18, 19) |
| $\frac{1}{\gamma^{vb}}$ | Average infectious period of breakthrough varicella | | | | 6 days ^C^ | | (19, 20) |
| $\frac{1}{\delta^{vb}}$ | Average waning period of zoster immunity after breakthrough varicella | | | | 81.3 years ^C^ | | Assumed equal to (15), [See section below](#_Estimation_of_boosting) ^D^ |
| **Herpes zoster reactivation** | | | | | | | |
| $\rho^{z}$ | Relative infectivity of HZ | | | | 7% | | (1) (23) |
| $\sigma_{j}$ | Wild HZ reactivation rate | | | |  | | Calibrated |
| $\chi$ | HZ reactivation rate on vaccine arms | | | | 1/6 | | (24) |
|  | HZ reactivation function parameters | | | | $\omega$: 0.063539  $\varphi$: 0.0977123  $\eta$: 2.34068  $\pi$: 0.0640124 | | Calibrated |
| $\frac{1}{\eta^{n}}$ | Average duration of HZ outbreak following natural varicella | | | | 28 days | | (1, 25, 26) |
| $\frac{1}{\eta^{vb}}$ | Average duration of HZ outbreak following breakthrough varicella | | | | 28 days | | (1) |
| $\frac{1}{\delta^{vv}}$ | Average waning period of exogenously boosted zoster immunity following successful varicella vaccination | | | | 81.3 years ^C^ | | (15),  [See section below](#_Estimation_of_boosting) ^D^ |
| *d_j_^phn^* | Average duration of PHN outbreak | | | | 9 months ^C^ | | (27) |
| **Exogenous boosting** | | | | | | | |
| $\zeta^{n}$ | Proportion of contacts leading to exogenous boosting after natural varicella | | | | 33.45% ^C^ | | (15),  [See section below](#_Estimation_of_boosting) ^D^ |
| $\zeta^{vb}$ | Proportion of contacts leading to exogenous boosting after breakthrough varicella | | | | 33.45% ^C^ | | (15),  [See section below](#_Estimation_of_boosting) ^D^ |
| $\zeta^{vv}$ | Proportion of contacts leading to exogenous boosting after varicella vaccination | | | | 33.45% ^C^ | | (15),  [See section below](#_Estimation_of_boosting) ^D^ |
| **Disease-related death** | | | | | | | |
|  |  | 0-5 | 5-15 | 15-45 | 45-65 | >65 |  |
| $d_{j}^{v}$ | Natural varicella infection-related death rate (deaths per 100,000 cases) | 1 | 1 | 9 | 73 | 689 ^C^ | (28) |
| $d_{j}^{vb}$ | Breakthrough varicella infection-related death rate (deaths per 100,000 cases) | 0 | 0 | 0 | 0 | 0 ^C^ | (29, 30, 31) |
| $d_{j}^{z}$ | HZ reactivation-related death rate (deaths per 100,000 cases) | 0 | 1 | 2 | 2 | 61 | (28) |

HZ, herpes zoster; PHN, postherpetic neuralgia.

^A^ Demographic inputs are age-structured and country-dependent and vary for each adaptation.

^B^ Force of mortality= -ln (1-annual probability of death).

^C^ These values were updated from Wolfson 2019.

^D^ See section on [Estimation of boosting proportion and duration of boosting](#_Estimation_of_boosting) in this S1 Text.

The system of ordinary differential equations describing the model is provided in the [Ordinary differential equations sub-section.](#_Ordinary_differential_equations)

The demographic model takes as inputs the population’s age distribution, fertility rate, and mortality rate (16, 32, 33, 34, 35). These rates were taken from country statistics provided by Statbank, Statistics Denmark. The contact mixing rate between age groups in the model influenced the likelihood that individuals in an age group become infected and was based on a study projecting the social contact rate in 152 countries (17), which combined information from the POLYMOD study of contacts between 2005 and 2006, and country-specific demographic data (36).

***Natural varicella***

Maternally-induced immunity following birth was calibrated from historical age-stratified varicella seroprevalence data and was determined to last 7.3 months (Table B).

Following this period, infants in the model became susceptible to varicella infection. Once exposed, the model separated the infection period into a latent and infectious period; during the infectious period, others may become infected at a rate governed by the contact mixing matrix.

For natural varicella infections, the average latent period was 14 days (18, 19), and the average infectious period was 7 days (19, 20, 21). The average latent period for natural varicella was based upon a 1929 study of the use of convalescent serum to prevent chickenpox (37). The latent period for natural varicella in the Control of Communicable Diseases Manual was also stated as 14-16 days (38).

***Breakthrough varicella***

The same average latent period (14 days) as natural varicella was used for breakthrough infection (19). However, the likelihood of a vaccinated individual infecting others following breakthrough varicella was reduced due to a shorter infectious period (6 days) and lower relative infectivity of breakthrough varicella as compared with natural varicella (19, 20). The average infectious period for natural and breakthrough varicella was based on a post-licensure effectiveness study conducted during a 1996 outbreak at a childcare center in the state of Georgia, USA (21). The relative infectivity of breakthrough varicella (50%) was based upon a population-based active surveillance study conducted from 1997-2001, which reported that vaccinated cases were half as contagious as unvaccinated cases (22). Brisson et al. (2000) also estimated 50% infectivity with breakthrough varicella based upon secondary attack rates from RCTs and the ratio of the numbers of lesions for vaccinated versus unvaccinated cases (detailed calculation provided in Brisson et al.) (18).

***Herpes zoster***

The relative contribution of HZ to varicella transmission (7%) was estimated by Ferguson et al. (1996) (23) using a stochastic SIR model and was used in models by Schuette and Hethcote (1). The reduction factor of the HZ reactivation rate for vaccinees (1/6) was taken from a multi-country model of varicella zoster virus transmission and reactivation applied to Italy, Finland, and the UK (24). The average duration of an uncomplicated HZ outbreak following breakthrough varicella (28 days) was taken from a case series of HZ in general practice in New South Wales, Australia (25) and was used in both Garnett et al (1996) (26) and Schuette Hethcote (1999) (1). The duration of postherpetic neuralgia (PHN) was 9 months (27). The PHN proportion was 2.9%, 5.2%, 10.2% and 12.5% for age ranges 50 to 59 years, 60 to 69 years, 70 to 79 years, and 80 years and above, respectively. It was assumed that there is no PHN below the age of 50 years (39).

A recently published self-controlled case series study of 9,604 UK adults with both household exposure to varicella and an episode of zoster showed that exposure to varicella is associated with a 33% reduction in risk of zoster over 20 years, suggesting that the impact of exogenous boosting may be lower than predicted in previous models (15). Another database study examining 20 years of real-world experience with UVV in the US found that the transient increase in HZ incidence predicted by the exogenous boosting hypothesis was not observed (40). Annual rates of change in HZ incidence were determined in an interrupted time series regression analysis for 1991-1995, 1996-2006, and 2007-2016, corresponding to pre-vaccination, one-dose vaccination, and two-dose vaccination periods, respectively. The annual incidence of HZ in adults increased at approximately the same rate in the years before and after implementing the varicella vaccination program. However, HZ incidence plateaued in adults beginning in 2012–2016, in contrast to the increases that would be expected under the exogenous boosting hypothesis. The increase in HZ incidence before implementing the varicella vaccination program in the US may be attributed to historic demographic changes that were not considered by previous models (41). Models that consider the interplay between exogenous boosting and historic demographic changes may more accurately predict the impact of varicella vaccination programs on HZ incidence. A population-based study in the US showed that approximately 38 in 100,000 children vaccinated against varicella developed HZ per year, compared to 170 per 100,000 unvaccinated children demonstrating the benefits of UVV on reducing the incidence of pediatric HZ (42).

Additional parameters related to HZ, including the waning period following natural or breakthrough varicella (81.3 years) and the proportion of contacts leading to exogenous boosting (33.45%), were calculated based on estimates of the relative incidence of HZ in the 20 years after exposure to a child with varicella in the household compared with baseline time from Forbes et al. (2020), a self-controlled case series study conducted using Clinical Practice Research Datalink (CPRD) with 9,604 adults (15). A simple model was developed to project HZ incidence, with parameters of interest calibrated to align with the data from Forbes (see Estimation of boosting proportion and duration of boosting subsection below for details) (43).

## Force of infection

The rate at which susceptible persons acquire infection was given by a time- and age-dependent force of infection, $\lambda_{j}(t)$, where $j$ is the age group of the susceptible person. The force of infection was computed using an empirical contact matrix for Denmark (17). As available data suggests, there is substantial transmission within households and the greater community (44); therefore, the all-location matrix was used.

The matrix was $16\times16$, using 15 five-year age groups for younger than 75 and having a single age group for persons over 75. These 16 age groups corresponded to one or more of the 49 age groups in the model, as given in Table C.

Table C. Mapping between data and model age groups

| **Contact matrix age group (**$\boldsymbol{J)}$ | **Age range** | **Model age groups contained (**$\boldsymbol{j)}$ |
| --- | --- | --- |
| 1 | [0,5) | 1-19 |
| 2 | [5,10) | 20-24 |
| 3 | [10,15) | 25-29 |
| 4 | [15,20) | 30-34 |
| 5 | [20,25) | 35 |
| 6 | [25,30) | 36 |
| 7 | [30,35) | 37 |
| 8 | [35,40) | 38 |
| 9 | [40,45) | 39 |
| 10 | [45,50) | 40 |
| 11 | [50,55) | 41 |
| 12 | [55,60) | 42 |
| 13 | [60,65) | 43 |
| 14 | [65,70) | 44 |
| 15 | [70,75) | 45 |
| 16 | >75 | 46-49 |

The convention that age groups from the model have lowercase indices $j, k, \ldots$, while age groups from the contact matrix have uppercase indices $J, K,\ldots$, was introduced here.

Rates of contact were distributed across the age strata in the dynamic transmission model proportional to their population distribution, assumed to be uniform within the five-year age groups; that is, if model age groups $j$ and $k$ are contained in contact matrix age groups $J$ and $K$, repesctively, the number of contacts people in age group $j$ have with people in age group $k$ is given by

$$c_{j,k}=\frac{c_{JK}n_{k}}{\sum_{k^{'}\in K} n_{k^{'}}}$$

where $n_{j}$ is the population of age group j. Thus, if $rr_{j}$ is the age-specific probability of a susceptible person in age group $j$ becoming infected, the force of infection is given by

$$\lambda_{j}\left( t \right)=rr_{j}\sum_{K} \sum_{k\in K} c_{J,K}\frac{n_{k}}{\sum_{k^{'}\in K} n_{k^{'}}} \left( i_{k\left( t \right)}+\rho^{v} ivb_{k}\left( t \right)+\rho^{z}\left( z_{k}\left( t \right)+zwild_{k}\left( t \right)+zvacc_{k}\left( t \right) \right) \right)$$

## Vaccination

Table D provides details on vaccine properties. The first dose take rate is defined as the rate at which a complete immunological response is induced following the first dose. Similarly, the second dose take rate is when a complete immunological response is induced following the second dose if the first dose did not provide complete immunity. The vaccine failure rate is the proportion of individuals who did not seroconvert within 42 days of vaccine administration.

Table D. Vaccine properties

| **Parameter** | **Definition** | **Dose** | **MSD vaccines** | **GSK vaccines** | **Source** |
| --- | --- | --- | --- | --- | --- |
| $1-P$ | Vaccine failure rate | 1^st^ & 2^nd^ | 4% | 5% | (45, 46) |
| $T_{l}$ | Dose take rate | 1^st^ | 90.3% | 61.7% | (7) |
|  |  | 2^nd^ | 69.0% | 83.4% | (7) |
|  |  | 1^st^ & 2^nd^ | 97% | 93.8% | (7) |
| $\frac{1}{\sigma^{v}}$ | Average duration of temporary protection against varicella after dose | 1^st^ & 2^nd^ | 1.2 years | 0.9 years | (7) |
| $\frac{1}{\pi_{l}}$ | Average waning period of high HZ immunity following varicella vaccination | 1^st^ | 81.3 years | 81.3 years | (15),  [See section below](#_Estimation_of_boosting) ^D^ |
|  |  | 2^nd^ | 81.3 years | 81.3 years |  |

HZ, herpes zoster.

Thus, 90.3% and 61.7% of the cohorts were assumed to gain “permanent” protection after the 1st dose of MSD and GSK vaccines, respectively, compared to a total of 97.0% and 93.8% after 2 doses, respectively. These vaccine “take” values, used as inputs in our model, are slightly different from the vaccine “efficacy” reported from these randomized controlled trials (1 dose: V-MSD: 94.4%, V-GSK: 67.2%; 2 dose: V-MSD: 98.3%, MMRV-GSK 95.4%) (8, 9) but are derived from the same 10-year randomized clinical trial data on all breakthrough infections, irrespective of severity, and fitted to a deterministic compartmental infectious disease model (7).

In our study we assumed the same efficacy of varicella vaccine for the monovalent and the varicella component of the quadrivalent formulations by the same company (47, 48).

## Estimation of boosting proportion and duration of boosting

Forbes estimated the relative incidence of HZ in the 20 years after exposure to a child with varicella in the household compared with baseline time (15). Using data from Forbes et al (15) (see Table E), we designed a simple model which attempts to estimate two parameters of the natural history of zoster vis-à-vis the proportion of individuals been boosted (ζ) and the duration of boosting (1/δn).

Table E. Incidence ratio of herpes zoster following household exposure to a child with varicella

| **Post-exposure risk period (years)** | **Mid-point (years)** | **Incidence ratio** |
| --- | --- | --- |
| 0–<2 | 1 | 0.67 |
| 2–<5 | 3.5 | 0.69 |
| 5–<10 | 7.5 | 0.69 |
| 10–20 | 15 | 0.73 |

HZ, herpes zoster.

Following recovery from varicella infection, individuals acquired a temporary immunity to zoster (r). Once immunity to zoster waned, individuals became susceptible to zoster (*w*) at a rate δn. Individuals could have a reactivation episode at rate *σ*. The simple model is given below as

$$r^{'}\left( t \right)=-\delta n r\left( t \right),$$

$$w^{'}(t)=\delta n r(t)-\sigma w(t),$$

$$r(0)=\zeta, w(0)=1-\zeta$$

The simple model assumed mortality and aging have little influence on boosting and its duration. The goal was to use the simple model to compute the analytical incidence ratio and fit the Forbes data (15). The solution for the number of individuals susceptible to zoster when boosting is

$$w\left( t,\zeta\right)=\frac{(\delta n-(1-\zeta)\sigma)ⅇ^{-\sigma t}-\delta n\zetaⅇ^{-\delta nt}}{\delta n-\sigma}.$$

The number of individuals susceptible to zoster without boosting is

$$w\left( t,0 \right)=ⅇ^{-\sigma t}.$$

The model incidence ratio was obtained by dividing the zoster incidence when boosting is active by zoster incidence without boosting. The model incidence ratio is given by

$$\frac{\delta n-(1-\zeta)\sigma-ⅇ^{t\left( -\delta n+\sigma\right)}\delta n\zeta}{\delta n-\sigma}.$$

Using reactivation rate $\sigma=ⅇ^{-a\phi}\omega+\frac{a^{\eta}\pi}{100000}$ and the associated values from Brisson (2002)(49), i.e., $\pi=1.06,\eta=1.91,\omega=0.11,\phi=0.17$ and $a=38.3$, the estimated average value for $\sigma$ over the next 20 years was $\sigma=0.0177$. Next, using the least-square method, the values $\delta n=0.012299$ and $\zeta=0.334499$ were obtained by minimizing the squared error between the model incidence ratio and the incidence ratio provided in the Forbes paper (also provided in Table E). Hence duration of boosting was 1/0.012299= 81.3 years. The least-square fit is depicted below in Fig B.

Fig B. Least-square fit of the incidence ratio


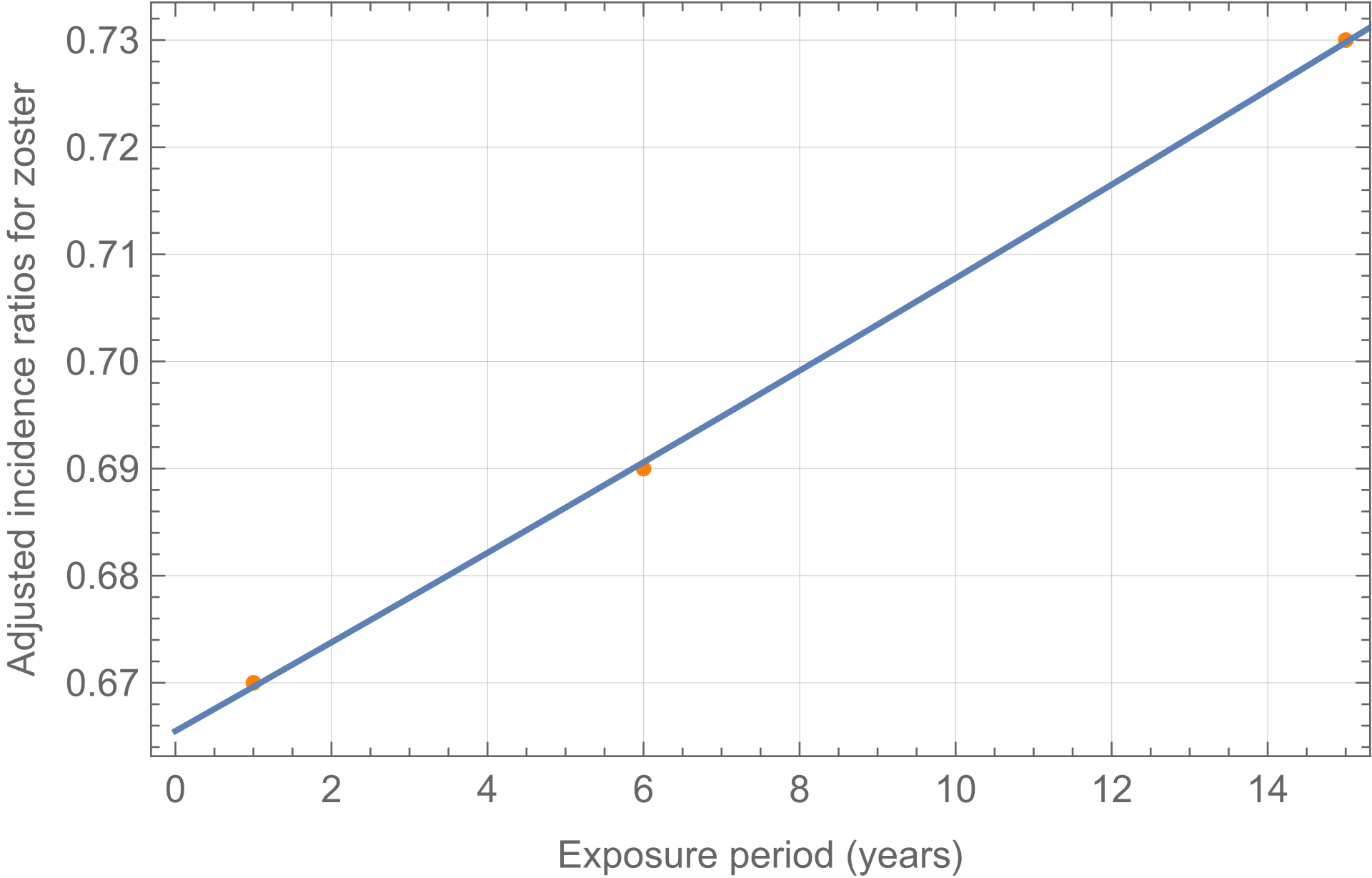


## Ordinary differential equations

$$\frac{{dm}_{j}}{dt}= B^{m}\left( t \right)\delta_{1,j}+d_{j-1}\left( {1-\delta}_{1,j} \right)\left( 1-\theta_{j}^{p}\left( t \right)-\theta_{j}^{c}\left( t \right)-\theta_{j}^{s}\left( t \right) \right)m_{j-1}\left( t \right) -\left( d_{j}+\omega^{m}+\mu_{j} \right)m_{j}\left( t \right).$$

$$\frac{{ds}_{j}}{dt}= B^{s}\left( t \right)\delta_{1,j}+ \omega^{m}m_{j}\left( t \right)+d_{j-1}\left( 1-\delta_{1,j} \right)\left( 1-\theta_{j}^{p}\left( t \right)-\theta_{j}^{c}\left( t \right)-\theta_{j}^{s}\left( t \right) \right)s_{j-1}\left( t \right) -\left( d_{j}+\mu_{j}+\lambda_{j} \right)s_{j}\left( t \right).$$

$$\frac{{de}_{j}}{dt}= \lambda_{j}s_{j}\left( t \right)+d_{j-1}\left( {1-\delta}_{1,j} \right)\left( 1-\theta_{j}^{p}\left( t \right)-\theta_{j}^{c}\left( t \right)-\theta_{j}^{s}\left( t \right) \right)e_{j-1}\left( t \right) -\left( d_{j}+\mu_{j}+\epsilon^{n} \right)e_{j}\left( t \right).$$

$$\frac{{di}_{j}}{dt}= d_{j-1}\left( {1-\delta}_{1,j} \right)i_{j-1}\left( t \right)+\epsilon^{n}\left( e_{j}\left( t \right)+ e_{j,1}\left( t \right)+e_{j,2}\left( t \right) \right)- \left( d_{j}+\mu_{j}+\gamma^{n}+d_{j}^{v} \right)i_{j}\left( t \right).$$

$$\frac{{dr}_{j}}{dt}= d_{j-1}\left( {1-\delta}_{1,j} \right)r_{j-1}\left( t \right) +\left( {\xi^{n}}_{j}+{\zeta^{n}}_{j}\lambda_{j} \right)w_{j}\left( t \right)+ \gamma^{n}i_{j}\left( t \right)-\left( d_{j}+\mu_{j}+\delta^{n} \right)r_{j}\left( t \right).$$

$$\frac{{dw}_{j}}{dt}=d_{j-1}\left( {1-\delta}_{1,j} \right)w_{j-1}\left( t \right)+\delta^{n}r_{j}\left( t \right)-\left( d_{j}+\mu_{j}+\sigma_{j}+{\xi^{n}}_{j}+{\zeta^{n}}_{j}\lambda_{j} \right)w_{j}\left( t \right).$$

$$\frac{{dv}_{j,1}}{dt}=d_{j-1}\left( {1-\delta}_{1,j} \right)v_{j-1,1}\left( t \right)+d_{j-1}\left( {1-\delta}_{1,j} \right)\left[ \left( \theta_{j}^{p}\left( t \right)+\theta_{j}^{c}\left( t \right)+\theta_{j}^{s}\left( t \right) \right)P T_{1}\left( m_{j-1}\left( t \right)+s_{j-1}\left( t \right) \right)-\left( \theta_{j}^{b}\left( t \right)+\theta_{j}^{d}\left( t \right) \right)v_{j-1,1}\left( t \right) \right]-\left( d_{j}+\mu_{j}+\pi_{1}+k_{1}\lambda_{j}\left( t \right) \right)v_{j,1}\left( t \right).$$

$$\frac{{dv}_{j,2}}{dt}=d_{j-1}\left( {1-\delta}_{1,j} \right)v_{j-1,2}\left( t \right)+d_{j-1}\left( {1-\delta}_{1,j} \right)\left( \theta_{j}^{b}\left( t \right)+\theta_{j}^{d}\left( t \right) \right)\left[ v_{j-1,1}\left( t \right)+P T_{1}s_{j-1,1}\left( t \right)+T_{2}\left( {vq}_{j-1,1}\left( t \right)+{vs}_{j-1,1}\left( t \right) \right) \right]-\left( d_{j}+\mu_{j}+\pi_{2}+k_{2}\lambda_{j}\left( t \right) \right)v_{j,2}\left( t \right).$$

$$\frac{{dvq}_{j,1}}{dt}=d_{j-1}\left( {1-\delta}_{1,j} \right){vq}_{j-1,1}\left( t \right)+d_{j-1}\left( {1-\delta}_{1,j} \right)\left[ P\left( 1-T_{1} \right)\left( \theta_{j}^{p}\left( t \right)+\theta_{j}^{c}\left( t \right)+\theta_{j}^{s}\left( t \right) \right)\left( m_{j-1}\left( t \right)+s_{j-1}\left( t \right) \right)-\left( \theta_{j}^{b}\left( t \right)+\theta_{j}^{d}\left( t \right) \right){vq}_{j-1,1}\left( t \right) \right]-\left( d_{j}+\mu_{j}+\sigma^{v} \right){vq}_{j,1}\left( t \right).$$

$$\frac{{dvq}_{j,2}}{dt}=d_{j-1}\left( {1-\delta}_{1,j} \right){vq}_{j-1,2}\left( t \right)+d_{j-1}\left( {1-\delta}_{1,j} \right)\left( \theta_{j}^{b}\left( t \right)+\theta_{j}^{d}\left( t \right) \right)\left[ P\left( 1-T_{1} \right)s_{j-1,1}\left( t \right)+\left( 1-T_{2} \right)\left( {vq}_{j-1,1}\left( t \right)+{vs}_{j-1,1}\left( t \right) \right) \right]-\left( d_{j}+\mu_{j}+\sigma^{v} \right){vq}_{j,2}\left( t \right).$$

$$\frac{{dvs}_{j,1}}{dt}= \sigma^{v}{vq}_{j,1}\left( t \right)+d_{j-1}\left( {1-\delta}_{1,j} \right)\left( 1-\theta_{j}^{b}\left( t \right)-\theta_{j}^{d}\left( t \right) \right){vs}_{j-1,1}\left( t \right)-\left( d_{j}+\mu_{j}+\lambda_{j}\left( t \right) \right){vs}_{j,1}\left( t \right).$$

$$\frac{{dvs}_{j,2}}{dt}= \sigma^{v}{vq}_{j,2}\left( t \right)+d_{j-1}\left( {1-\delta}_{1,j} \right){vs}_{j-1,2}\left( t \right)-\left( d_{j}+\mu_{j}+\lambda_{j}\left( t \right) \right){vs}_{j,2}\left( t \right).$$

$$\frac{{ds}_{j,1}}{dt}= d_{j-1}\left( {1-\delta}_{1,j} \right)s_{j-1,1}\left( t \right)+d_{j-1}\left( {1-\delta}_{1,j} \right)\left[ \left( 1-P \right)\left( \theta_{j}^{p}\left( t \right)+\theta_{j}^{c}\left( t \right)+\theta_{j}^{s}\left( t \right) \right)\left( m_{j-1}\left( t \right)+s_{j-1}\left( t \right) \right)-\left( \theta_{j}^{b}\left( t \right)+\theta_{j}^{d}\left( t \right) \right)s_{j-1,1}\left( t \right) \right]-\left( d_{j}+\mu_{j}+\lambda_{j}\left( t \right) \right)s_{j,1}\left( t \right).$$

$$\frac{{ds}_{j,2}}{dt}= d_{j-1}\left( {1-\delta}_{1,j} \right)s_{j-1,2}\left( t \right)+d_{j-1}\left( {1-\delta}_{1,j} \right)\left( 1-P \right)\left( \theta_{j}^{b}\left( t \right)+\theta_{j}^{d}\left( t \right) \right)s_{j-1,1}\left( t \right)-\left( d_{j}+\mu_{j}+\lambda_{j}\left( t \right) \right)s_{j,2}\left( t \right).$$

$$\frac{{de}_{j,1}}{dt}=d_{j-1}\left( {1-\delta}_{1,j} \right)e_{j-1,1}\left( t \right)+d_{j-1}\left( {1-\delta}_{1,j} \right)\left[ \left( \theta_{j}^{p}\left( t \right)+\theta_{j}^{c}\left( t \right)+\theta_{j}^{s}\left( t \right) \right)e_{j-1}\left( t \right)-\left( \theta_{j}^{b}\left( t \right)+\theta_{j}^{d}\left( t \right) \right)e_{j-1,1}\left( t \right) \right]+\lambda_{j}\left( t \right)s_{j,1}\left( t \right)- \left( d_{j}+\mu_{j}+\epsilon^{n} \right)e_{j,1}\left( t \right).$$

$$\frac{{de}_{j,2}}{dt}=d_{j-1}\left( {1-\delta}_{1,j} \right)e_{j-1,2}\left( t \right)+d_{j-1}\left( {1-\delta}_{1,j} \right)\left( \theta_{j}^{b}\left( t \right)+\theta_{j}^{d}\left( t \right) \right)e_{j-1,1}\left( t \right)+\lambda_{j}\left( t \right)s_{j,2}\left( t \right)- \left( d_{j}+\mu_{j}+\epsilon^{n} \right)e_{j,2}\left( t \right).$$

$$\frac{{drvv}_{j,1}}{dt}= d_{j-1}\left( {1-\delta}_{1,j} \right)\left( 1-\theta_{j}^{b}\left( t \right)-\theta_{j}^{d}\left( t \right) \right){rvv}_{j-1,1}\left( t \right)+{\xi^{vv}}_{j}{wvv}_{j,1}\left( t \right)+\lambda_{j}\left( t \right)\left[ k_{1}v_{j,1}\left( t \right)+{\zeta^{vv}}_{j}{wvv}_{j,1}\left( t \right) \right]-\left( d_{j}+\mu_{j}+\delta^{vv} \right){rvv}_{j,1}\left( t \right).$$

$$\frac{{drvv}_{j,2}}{dt}= d_{j-1}\left( {1-\delta}_{1,j} \right)\left[ \left( \theta_{j}^{b}\left( t \right)+\theta_{j}^{d}\left( t \right) \right){rvv}_{j-1,1}\left( t \right)+{rvv}_{j-1,2}\left( t \right) \right]+{\xi^{vv}}_{j}{wvv}_{j,2}\left( t \right)+\lambda_{j}\left( t \right)\left[ k_{2}v_{j,2}\left( t \right)+{\zeta^{vv}}_{j}{wvv}_{j,2}\left( t \right) \right]-\left( d_{j}+\mu_{j}+\delta^{vv} \right){rvv}_{j,2}\left( t \right).$$

$$\frac{{dwvv}_{j,1}}{dt}=d_{j-1}\left( {1-\delta}_{1,j} \right)\left( 1-\theta_{j}^{b}\left( t \right)-\theta_{j}^{d}\left( t \right) \right){wvv}_{j-1,1}\left( t \right)+\delta^{vv}{rvv}_{j,1}\left( t \right)+\pi_{1}v_{j,1}\left( t \right)-\left( d_{j}+\mu_{j}+\chi\sigma_{j}+{\xi^{vv}}_{j}+{\zeta^{vv}}_{j}\lambda_{j}\left( t \right) \right){wvv}_{j,1}\left( t \right).$$

$$\frac{{dwvv}_{j,2}}{dt}=d_{j-1}\left( {1-\delta}_{1,j} \right){wvv}_{j-1,2}\left( t \right)+d_{j-1}\left( {1-\delta}_{1,j} \right)\left( \theta_{j}^{b}\left( t \right)+\theta_{j}^{d}\left( t \right) \right){wvv}_{j-1,1}\left( t \right)+\delta^{vv}{rvv}_{j,2}\left( t \right)+\pi_{2}v_{j,2}\left( t \right)-\left( d_{j}+\mu_{j}+\chi\sigma_{j}+{\xi^{vv}}_{j}+{\zeta^{vv}}_{j}\lambda_{j}\left( t \right) \right){wvv}_{j,2}\left( t \right).$$

$$\frac{{devb}_{j,1}}{dt}= d_{j-1}\left( {1-\delta}_{1,j} \right)\left( 1-\theta_{j}^{b}\left( t \right)-\theta_{j}^{d}\left( t \right) \right){evb}_{j-1,1}\left( t \right)+\lambda_{j}\left( t \right){vs}_{j,1}\left( t \right)- \left( d_{j}+\mu_{j}+\epsilon^{vb} \right){evb}_{j,1}\left( t \right).$$

$$\frac{{devb}_{j,2}}{dt}= d_{j-1}\left( {1-\delta}_{1,j} \right){evb}_{j-1,2}\left( t \right)+\lambda_{j}\left( t \right){vs}_{j,2}\left( t \right)+d_{j-1}\left( {1-\delta}_{1,j} \right)\left( \theta_{j}^{b}\left( t \right)+\theta_{j}^{d}\left( t \right) \right){evb}_{j-1,1}\left( t \right)- \left( d_{j}+\mu_{j}+\epsilon^{vb} \right){evb}_{j,2}\left( t \right).$$

$$\frac{{divb}_{j}}{dt}= d_{j-1}\left( {1-\delta}_{1,j} \right){ivb}_{j-1}\left( t \right)+\epsilon^{vb}\left( {evb}_{j,1}\left( t \right)+{evb}_{j,2}\left( t \right) \right)-\left( d_{j}+\mu_{j}+\gamma^{vb}+d_{j}^{vb} \right){ivb}_{j}\left( t \right).$$

$$\frac{{drvb}_{j}}{dt}= d_{j-1}\left( {1-\delta}_{1,j} \right){rvb}_{j-1}\left( t \right)+\left( {\xi^{vb}}_{j}+{\zeta^{vb}}_{j}\lambda_{j}\left( t \right) \right){wvb}_{j}\left( t \right)+\gamma^{vb}{ivb}_{j}\left( t \right)-\left( d_{j}+\mu_{j}+\delta^{vb} \right){rvb}_{j}\left( t \right).$$

$$\frac{{dwvb}_{j}}{dt}= d_{j-1}\left( {1-\delta}_{1,j} \right)\left( 1-\theta_{j}^{z}\left( t \right) \right){wvb}_{j-1}\left( t \right)+\delta^{vb}{rvb}_{j}\left( t \right)-\left( d_{j}+\mu_{j}+\chi\sigma_{j}+{\xi^{vb}}_{j}+{\zeta^{vb}}_{j}\lambda_{j}\left( t \right) \right){wvb}_{j}\left( t \right).$$

$$\frac{{dz}_{j}}{dt}= \sigma_{j}w_{j}\left( t \right)+d_{j-1}\left( {1-\delta}_{1,j} \right)z_{j-1}\left( t \right)-\left( d_{j}+\mu_{j}+\eta^{n}+d_{j}^{z} \right)z_{j}\left( t \right).$$

$$\frac{{dzvacc}_{j}}{dt}=d_{j-1}\left( {1-\delta}_{1,j} \right){zvacc}_{j-1}\left( t \right)+\chi\sigma_{j}\left( {wvv}_{j,1}\left( t \right)+{wvv}_{j,2}\left( t \right) \right)-\left( d_{j}+\mu_{j}+\eta^{vv}+d_{j}^{z} \right){zvacc}_{j}\left( t \right).$$

$$\frac{{dzwild}_{j}}{dt}=d_{j-1}\left( {1-\delta}_{1,j} \right){zwild}_{j-1}\left( t \right)+\chi\sigma_{j}{wvb}_{j}\left( t \right)-\left( d_{j}+\mu_{j}+\eta^{vb}+d_{j}^{z} \right){zwild}_{j}\left( t \right).$$

$$\frac{{drz}_{j}}{dt}=d_{j-1}\left( {1-\delta}_{1,j} \right){rz}_{j-1}\left( t \right)+\eta^{n}z_{j}\left( t \right)+\eta^{vb}{zwild}_{j}\left( t \right)+\eta^{vv}{zvacc}_{j}\left( t \right)-\left( d_{j}+\mu_{j} \right){rz}_{j}\left( t \right).$$

$$\frac{{dndv}_{j}}{dt}=d_{j-1}\left( {1-\delta}_{1,j} \right){ndv}_{j-1}\left( t \right)+d_{j}^{v}i_{j}\left( t \right)+d_{j}^{vb}{ivb}_{j}\left( t \right)-\left( d_{j}+\mu_{j} \right){ndv}_{j}\left( t \right).$$

$$\frac{{dndz}_{j}}{dt}=d_{j-1}\left( {1-\delta}_{1,j} \right){ndz}_{j-1}\left( t \right)+d_{j}^{z}\left( z_{j}\left( t \right)+{zwild}_{j}\left( t \right)+{zvacc}_{j}\left( t \right) \right)-\left( d_{j}+\mu_{j} \right){ndz}_{j}\left( t \right).$$

$$\lambda_{j}\left( t \right)=\sum_{a} \beta_{j,a}\left( i_{a}\left( t \right)+\rho^{v}{ivb}^{a}\left( t \right)+\rho^{z}z_{a}\left( t \right)+\rho^{z}{zwild}_{a}\left( t \right)+\rho^{z}{zvacc}_{a}\left( t \right) \right).$$

$$B^{m}\left( t \right)=\sum_{j} f_{j}n_{j}- \sum_{j} \left( f_{j}s_{j}\left( t \right)+\sum_{l} f_{j}s_{j,l}\left( t \right) \right).$$

$$B^{s}\left( t \right)= \sum_{j} \left( f_{j}s_{j}\left( t \right)+\sum_{l} f_{j}s_{j,l}\left( t \right) \right).$$

**Definition of vaccination coverage**

No vaccination strategy:

$$\theta_{j}^{p}\left( t \right)=\theta_{j}^{c}\left( t \right)=\theta_{j}^{b}\left( t \right)= \theta_{j}^{d}\left( t \right)=0$$

2-dose short interval with catch-up:

- Primary vaccination at 12 months of age (j=5) starting at time=0
- Booster vaccination at 15 months of age (j=8) for children who received primary dose
- Primary catch-up vaccination at 2-12 years of age (j=17-26) in first year of UVV only (time=0)
- Booster catch-up vaccination at 3-13 years of age (j=18-27) in second year of UVV only for children who received primary catch-up vaccination

$$\begin{aligned} \theta_{j}^{p}\left( t \right)= \delta_{j,5}\phi^{p} t\geq0. \\ \theta_{j}^{c}\left( t \right)=\delta_{j,17-26}\phi^{c} 0\leq t\leq1. \\ \theta_{j}^{b}\left( t \right)=\left\{ \begin{matrix} 0 & t<3 months, \\ \delta_{j,8}\phi^{bs} & t\geq3 months. \end{matrix} \right. \\ \theta_{j}^{d}\left( t \right)=\delta_{j,18-27}\phi^{d} 1\leq t\leq2. \end{aligned}$$

2-dose medium interval with catch-up:

- Primary vaccination at 15 months of age (j=8) starting at time=0
- Booster vaccination at 48 months of age (j=19) for children who received primary dose
- Primary catch-up vaccination at 2-12 years of age (j=17-26) in first year of UVV only (time=0)
- Booster catch-up vaccination at 3-13 years of age (j=18-27) in second year of UVV only for children who received primary catch-up vaccination

$$\begin{aligned} \theta_{j}^{p}\left( t \right)= \delta_{j,8}\phi^{p} t\geq0. \\ \theta_{j}^{c}\left( t \right)=\delta_{j,17-26}\phi^{c} 0\leq t\leq1. \\ \theta_{j}^{b}\left( t \right)=\left\{ \begin{matrix} 0 & t<33 months, \\ \delta_{j,19}\phi^{bl} & t\geq33 months. \end{matrix} \right. \\ \theta_{j}^{d}\left( t \right)=\delta_{j,18-27}\phi^{d} 1\leq t\leq2. \end{aligned}$$

Zoster vaccination is excluded for all strategies:

$$\theta_{j}^{z}\left( t \right)= 0 t\geq0$$

The values of $\phi$ refer to the different coverage rates described in detail in the main text.

## References

1. Schuette MC, Hethcote HW. Modeling the effects of varicella vaccination programs on the incidence of chickenpox and shingles. Bulletin of mathematical biology. 1999;61(6):1031-64.

2. Wolfson LJ, Daniels VJ, Pillsbury M, Kurugöl Z, Yardimci C, Kyle J, et al. Cost-effectiveness analysis of universal varicella vaccination in Turkey using a dynamic transmission model. PLoS One. 2019;14(8):e0220921.

3. Azzari C, Baldo V, Giuffrida S, Gani R, O'Brien E, Alimenti C, et al. The Cost-Effectiveness of Universal Varicella Vaccination in Italy: A Model-Based Assessment of Vaccination Strategies. ClinicoEconomics and outcomes research : CEOR. 2020;12:273-83.

4. Graham J, Wolfson LJ, Kyle J, Bolde-Villarreal CP, Guarneros-DeRegil DB, Monsanto H, et al. Budget impact analysis of multiple varicella vaccination strategies: a Mexico perspective. Hum Vaccin Immunother. 2020;16(4):886-94.

5. Heininger U, Pillsbury M, Samant S, Lienert F, Guggisberg P, Gani R, et al. Health Impact and Cost-effectiveness Assessment for the Introduction of Universal Varicella Vaccination in Switzerland. The Pediatric infectious disease journal. 2021;40(6):e217-e21.

6. Pawaskar M, Burgess C, Pillsbury M, Wisløff T, Flem E. Clinical and economic impact of universal varicella vaccination in Norway: A modeling study. PLoS One. 2021;16(7):e0254080.

7. Pillsbury M, Carias C, Samant S, Greenberg D, Pawaskar M. Comparison of performance of varicella vaccines via infectious disease modeling. Vaccine. 2022.

8. Kuter B, Matthews H, Shinefield H, Black S, Dennehy P, Watson B, et al. Ten year follow-up of healthy children who received one or two injections of varicella vaccine. The Pediatric infectious disease journal. 2004;23(2):132-7.

9. Povey M, Henry O, Riise Bergsaker MA, Chlibek R, Esposito S, Flodmark CE, et al. Protection against varicella with two doses of combined measles-mumps-rubella-varicella vaccine or one dose of monovalent varicella vaccine: 10-year follow-up of a phase 3 multicentre, observer-blind, randomised, controlled trial. Lancet Infect Dis. 2019;19(3):287-97.

10. Guzzetta G, Poletti P, Merler S, Manfredi P. The Epidemiology of Herpes Zoster After Varicella Immunization Under Different Biological Hypotheses: Perspectives From Mathematical Modeling. Am J Epidemiol. 2016;183(8):765-73.

11. Erhverv D. Samfundsøkonomisk potentiale ved vaccination 2020 [Available from: <https://www.danskerhverv.dk/siteassets/mediafolder/dokumenter/01-analyser/analysenotater-2020/samfundsokonomisk-potentiale-ved-vaccination.pdf>.

12. Ogunjimi B, Van Damme P, Beutels P. Herpes Zoster Risk Reduction through Exposure to Chickenpox Patients: A Systematic Multidisciplinary Review. PLoS One. 2013;8(6):e66485.

13. Talbird SE, La EM, Mauskopf J, Altland A, Daniels V, Wolfson LJ. Understanding the role of exogenous boosting in modeling varicella vaccination. Expert Rev Vaccines. 2018;17(11):1021-35.

14. Damm O, Ultsch B, Horn J, Mikolajczyk RT, Greiner W, Wichmann O. Systematic review of models assessing the economic value of routine varicella and herpes zoster vaccination in high-income countries. BMC public health. 2015;15:533.

15. Forbes H, Douglas I, Finn A, Breuer J, Bhaskaran K, Smeeth L, et al. Risk of herpes zoster after exposure to varicella to explore the exogenous boosting hypothesis: self controlled case series study using UK electronic healthcare data. BMJ. 2020;368:l6987.

16. Statbank Denmark. Statistics Denmark [Available from: <https://www.statbank.dk/statbank5a/>.

17. Prem K, Cook AR, Jit M. Projecting social contact matrices in 152 countries using contact surveys and demographic data. PLoS computational biology. 2017;13(9):e1005697.

18. Brisson M, Edmunds WJ, Gay NJ, Law B, De Serres G. Modelling the impact of immunization on the epidemiology of varicella zoster virus. Epidemiology and infection. 2000;125(3):651-69.

19. Marin M, Bialek SR. Varicella/Herpes Zoster. Control of Communicable Diseases Manual. 16th Edition. Washington, DC: American Public Health Association; 2015.

20. Bernstein HH, Rothstein EP, Watson BM, Reisinger KS, Blatter MM, Wellman CO, et al. Clinical survey of natural varicella compared with breakthrough varicella after immunization with live attenuated Oka/Merck varicella vaccine. Pediatrics. 1993;92(6):833-7.

21. Izurieta HS, Strebel PM, Blake PA. Postlicensure effectiveness of varicella vaccine during an outbreak in a child care center. JAMA. 1997;278(18):1495-9.

22. Seward JF, Zhang JX, Maupin TJ, Mascola L, Jumaan AO. Contagiousness of varicella in vaccinated cases: a household contact study. JAMA. 2004;292(6):704-8.

23. Ferguson NM, Anderson RM, Garnett GP. Mass vaccination to control chickenpox: the influence of zoster. Proceedings of the National Academy of Sciences of the United States of America. 1996;93(14):7231-5.

24. Poletti P, Melegaro A, Ajelli M, Del Fava E, Guzzetta G, Faustini L, et al. Perspectives on the impact of varicella immunization on herpes zoster. A model-based evaluation from three European countries. PLoS One. 2013;8(4):e60732.

25. Trollor J. Herpes zoster in general practice. Australian family physician. 1987;16(8):1133, 7-40.

26. Garnett GP, Grenfell BT. The epidemiology of varicella-zoster virus infections: a mathematical model. Epidemiology and infection. 1992;108(3):495-511.

27. Gauthier A, Breuer J, Carrington D, Martin M, Rémy V. Epidemiology and cost of herpes zoster and post-herpetic neuralgia in the United Kingdom. Epidemiology and infection. 2009;137(1):38-47.

28. Brisson M, Edmunds WJ. Varicella vaccination in England and Wales: cost-utility analysis. Archives of disease in childhood. 2003;88(10):862-9.

29. Akpo EIH, Cristeau O, Hunjan M, Casabona G. Epidemiological Impact and Cost-Effectiveness of Varicella Vaccination Strategies in the United Kingdom. Clin Infect Dis. 2021;73(11):e3617-e26.

30. Melegaro A, Marziano V, Del Fava E, Poletti P, Tirani M, Rizzo C, et al. The impact of demographic changes, exogenous boosting and new vaccination policies on varicella and herpes zoster in Italy: a modelling and cost-effectiveness study. BMC medicine. 2018;16(1):117.

31. Leung J, Bialek SR, Marin M. Trends in varicella mortality in the United States: Data from vital statistics and the national surveillance system. Hum Vaccin Immunother. 2015;11(3):662-8.

32. Statbank Denmark. FOD 33: Age-specific fertility rates (single year), total fertility rate (ages 15-49), and gross and net reproduction rates [updated April 19, 2022. Available from: <https://www.statbank.dk/statbank5a/SelectVarVal/Define.asp?Maintable=FOD33&PLanguage=1>.

33. Statbank Denmark. FOD207: Deaths by region, age and sex [updated Apriil 19, 2022. Available from: <https://www.statbank.dk/statbank5a/SelectVarVal/Define.asp?Maintable=FOD207&PLanguage=1>.

34. Statbank Denmark. [updated April 19, 2022. Available from: <https://www.statbank.dk/statbank5a/selectvarval/define.asp?PLanguage=1&subword=tabsel&MainTable=FOLK1A&PXSId=199114&tablestyle=&ST=SD&buttons=0>.

35. Statistics Norway. Statbank: Births 2021 [Available from: <https://www.ssb.no/statbank/table/09745/tableViewLayout1/>.

36. Mossong J, Hens N, Jit M, Beutels P, Auranen K, Mikolajczyk R, et al. Social contacts and mixing patterns relevant to the spread of infectious diseases. PLoS Med. 2008;5(3):e74.

37. Gordon JE, Meader FM. The period of infectivity an serum prevention of chickenpox. JAMA. 1929;93:2013-15.

38. Heymann DL. Control of communicable diseases manual: an official report of the American Public Health Association. American Public Health Association; 2015.

39. Klein NP, Bartlett J, Fireman B, Marks MA, Hansen J, Lewis E, et al. Long-term effectiveness of zoster vaccine live for postherpetic neuralgia prevention. Vaccine. 2019;37(36):5422-7.

40. Weinmann S, Chun C, Schmid DS, Roberts M, Vandermeer M, Riedlinger K, et al. Incidence and clinical characteristics of herpes zoster among children in the varicella vaccine era, 2005-2009. J Infect Dis. 2013;208(11):1859-68.

41. Harpaz R. Do varicella vaccination programs change the epidemiology of herpes zoster? A comprehensive review, with focus on the United States. Expert Rev Vaccines. 2019;18(8):793-811.

42. Weinmann S, Naleway AL, Koppolu P, Baxter R, Belongia EA, Hambidge SJ, et al. Incidence of Herpes Zoster Among Children: 2003-2014. Pediatrics. 2019;144(1).

43. Kujawski SA, Burgess C, Agi O, Attias-Geva Z, Pillsbury M, Greenberg D, et al. The health and economic impact of switching vaccines in universal varicella vaccination programs using a dynamic transmission model: An Israel case study. Hum Vaccin Immunother. 2022;18(6):2124784.

44. Yawn BP, Yawn RA, Lydick E. Community impact of childhood varicella infections. J Pediatr. 1997;130(5):759-65.

45. van Hoek AJ, Melegaro A, Zagheni E, Edmunds WJ, Gay N. Modelling the impact of a combined varicella and zoster vaccination programme on the epidemiology of varicella zoster virus in England. Vaccine. 2011;29(13):2411-20.

46. Ouwens MJ, Littlewood KJ, Sauboin C, Téhard B, Denis F, Boëlle PY, et al. The impact of 2-dose routine measles, mumps, rubella, and varicella vaccination in France on the epidemiology of varicella and zoster using a dynamic model with an empirical contact matrix. Clinical therapeutics. 2015;37(4):816-29 e10.

47. Annex I: Summary of Product Characteristics (ProQuad): European Medicines Agency; [Available from: <https://www.ema.europa.eu/en/documents/product-information/proquad-epar-product-information_en.pdf>.

48. Inc. G. Product Monograph: Priorix-Tetra Mississauga, Ontario2019 [Available from: <https://ca.gsk.com/media/6253/priorix-tetra.pdf>.

49. Brisson M, Gay NJ, Edmunds WJ, Andrews NJ. Exposure to varicella boosts immunity to herpes-zoster: implications for mass vaccination against chickenpox. Vaccine. 2002;20(19-20):2500-7.
