# Supplementary material for: Universal varicella vaccination in Denmark: Modeling public health impact, age-shift, and cost-effectiveness
Source: PLOS Glob Public Health. 2023 Apr 5;3(4):e0001743. doi: 10.1371/journal.pgph.0001743 (PMC10075481; doi:10.1371/journal.pgph.0001743)
Supplement: S2 Text — (DOCX) [file pgph.0001743.s002.docx]

# S2 Text: Model inputs specific for Denmark adaptation

- [Vaccination schedule](#_Vaccination_schedule)
- [Vaccination cost](#_Vaccination_cost)
  - Table A. Varicella vaccination costs per dose
- [Denmark-specific utility values](#_Denmark-specific_utility_values)
  - Table B. Age-specific health state utility values
- [Health economic parameters](#_Health_economic_parameters)
  - Table C. Inputs for healthcare resource utilization and costs in the model
  - Table D. Varicella resource utilization
- [Denmark model calibration](#_Denmark_model_calibration)
  - Fig A. Observed and fitted A) varicella seroprevalence and B) herpes zoster incidence
- [References](#_References)

## Vaccination schedule

For children receiving their first vaccine dose at 12 months, the second dose was administered at 15 months (short interval); for children receiving their first dose at 15 months, the second dose was administered at 48 months (medium interval).

Catch-up vaccination: Children who were 2-12 years at time of UVV introduction are eligible for catch-up vaccination with 2 doses of monovalent vaccine. The first catch-up dose was given in the first year of UVV introduction with the second catch-up dose given either at the recommended age of the second routine dose or 1 year after the first catch-up dose, whichever is later.

## Vaccination cost

The direct cost of vaccination was the sum of the costs of vaccine acquisition and administration (Table A). The acquisition cost of monovalent vaccines was the average of list prices for countries in the International Reference Pricing system (Sweden, Norway, Finland, UK, The Netherlands, Belgium, Germany, Ireland and Austria) for which data was available [1]. The acquisition cost of the varicella component of the quadrivalent vaccines was calculated as the list price of the MMRV vaccine minus the list price of the MMR vaccine in Germany (the only country with list prices for both the MMR and MMRV vaccines from both companies). The administration cost for the monovalent varicella vaccines was assumed to be same as that for the MMR vaccine. There was no additional administration cost for the varicella component of the quadrivalent vaccines. No travel or parent productivity loss was included in the cost of administration, since it was assumed that the vaccination would be given at the time of a routinely scheduled well-visit.

Table A. Varicella vaccination costs per dose

| **Vaccine** | **Acquisition cost** | **Administration cost** | **Total** |
| --- | --- | --- | --- |
| V-MSD | €39.25 | €6.24 | €45.49 |
| V-GSK | €40.63 | €6.24 | €46.87 |
| Varicella component of MMRV-MSD | €44.41 | €0.00 | €44.41 |
| Varicella component of MMRV-GSK | €47.72 | €0.00 | €47.72 |

## Denmark-specific utility values

Table B. Age-specific health state utility values

| **Parameter** | **Health state** | **Age (in years)** | | | | | | | | | | | **Source** |
| --- | --- | --- | --- | --- | --- | --- | --- | --- | --- | --- | --- | --- | --- |
|  |  | **0-15** | **15-17** | **18-24** | **25-34** | **35-44** | **45-54** | **55-64** | **65-69** | **70-75** | | **>75** |  |
| $q_{j}^{h}$ | Healthy ^A^ | 1.000 | | 0.885 | 0.884 | 0.845 | 0.822 | 0.799 | 0.766 | | | 0.691 | Szende 2014 [2] |
| $q_{j}^{v}$ | Natural varicella | 0.791 | 0.739 | | | | | | | | | | Brisson 2003 [3] |
| $q_{j}^{vb}$ | Breakthrough varicella | 0.939 | | | | | | | | | | |  |
| $q_{j}^{z}$ | Uncomplicated herpes zoster | 0.870 | | | | | | | | | 0.844 | | Pellissier 2007 [4] |
| $q_{j}^{zv}$ | Uncomplicated herpes zoster breakthrough | 0.870 | | | | | | | | | 0.844 | |  |
| $q_{j}^{phn}$ | Postherpetic neuralgia | 0.657 | | | | | | | | | | | Oster 2005 [5] |

^A^ These values were updated from Wolfson 2019.

## Health economic parameters

Denmark-specific healthcare resource use data were either not available or of inadequate resolution to be used in the model. Hence, most of the varicella-related outpatient and inpatient resource utilization parameters were taken from a recent study set in Sweden [6]. Like Denmark, Sweden has neither varicella nor HZ vaccination in its national immunization program [6]. We combined primary care and specialist care percentages from the Swedish study to approximate outpatient care utilization. Productivity loss associated with varicella was assumed to be zero for cases older than 65 years [6]. HZ-related productivity loss was assumed to be zero for age groups 0-45 years and >65 years. Healthcare resource utilization parameter values are shown in Table C.

Table C. Inputs for healthcare resource utilization and costs in the model

| **Varicella-related HCRU and costs** | | |
| --- | --- | --- |
| % of varicella cases seeking outpatient care | See Table D below for natural varicella | Wolff 2021 [6] |
| Mean number of outpatient visits per varicella case | 2 visits for natural varicella; 1 visit for breakthrough varicella | Assumption |
| Cost of a single outpatient visit | €19.67 | [7] |
| % of varicella cases requiring hospital | See Table D below for natural varicella; Breakthrough varicella was assumed to be 20% of natural varicella | Wolff 2021 [6] |
| Average hospital length of stay (in days) | See Table D below | Wolff 2021 [6] |
| Daily cost of hospitalization | €229.37 per day ^A^ | WHO-choice [8] |
| **HZ-related HCRU and costs** | | |
| % of HZ cases that are uncomplicated | <50 years: 100%  50-59 years: 97.1%  60-69 years: 94.8%  70-79 years:89.8%  ≥80 years: 87.5% | Klein 2019 [9] |
| % of uncomplicated HZ cases requiring medical care | <45 years: 0%  ≥45 years: 100% | Haugnes 2019 [10] |
| Cost of treating an uncomplicated HZ case | €3,995.00 ^B^ | Haugnes 2019 [10] |
| % of HZ cases that develop PHN | <50 years: 0%  50-59 years:2.9 %  60-69 years: 5.2%  70-79 years: 12.5%  ≥80 years: 12.5% | Klein 2019 [9] |
| % of PHN cases requiring medical care | <45 years: 0%  ≥45 years: 100% | Assumption |
| Cost of treating a PHN case | €5,681.00 ^B^ | Haugnes 2019 [10] |
| Productivity loss | | |
| Average cost per workday | €181.88 ^C^ |  |
| Workdays lost for varicella patients and caregivers | See Table D below; Productivity loss for ≥65 assumed to be zero |  |
| Workdays lost per uncomplicated HZ case | 0-45 years: 0  45 to <65 years: 0.30  ≥65: 0 | Adapted by Heininger 2021 [11] |
| Workdays lost per PHN case | 0-45 years: 0  45 to <65 years: 1.90  ≥65: 0 | Adapted by Heininger 2021 [11] |

HCRU, healthcare resource utilization; HZ, herpes zoster; PHN, postherpetic neuralgia.

^A^ Mean hospital cost per day based on WHO-choice in 2005 for Denmark (primary cost per bed day by hospital level), inflated to 2020 price [8].

^B^ “Zoster without complications” €3890 (2017 costs) updated to €3,995; “Zoster with other complications” are €5,532 (2017 costs) updated to €5,681.

^C^ Average cost per workday converted from 1,352.24 Danish Krone to Euros.

Table D. Varicella resource utilization

| **Age group ^A^** | **Primary care % ^A^** | **Specialist care % ^A^** | **Outpatient resource utilization % ^B^** | **Hospitalization %** | **Average number of days hospitalized ^A^** | **Average number of days with productivity loss ^A^** |
| --- | --- | --- | --- | --- | --- | --- |
| 0 | 3 | 1.9 | 4.9 | 0.2 | 3.7 | 2.40 |
| 1 | 13 | 2.5 | 15.5 | 0.4 | 3.0 | 3.57 |
| 2 | 17 | 2.3 | 19.3 | 0.4 | 3.7 | 3.61 |
| 3 | 8 | 1.0 | 9.0 | 0.2 | 4.3 | 3.52 |
| 4 | 6 | 0.7 | 6.7 | 0.1 | 3.3 | 3.50 |
| 5 | 7 | 0.8 | 7.8 | 0.2 | 3.9 | 3.46 |
| 6 | 6 | 0.7 | 6.7 | 0.1 | 4.2 | 3.46 |
| 7 | 7 | 0.9 | 7.9 | 0.2 | 3.9 ^C^ | 3.48 |
| 8 | 6 | 0.6 | 6.6 | 0.1 | 3.9 ^C^ | 3.44 |
| 9 | 5 | 0.8 | 5.8 | 0.1 | 3.9 ^C^ | 3.58 |
| 10 | 7 | 1.1 | 8.1 | 1.0 | 3.9 | 3.45 |
| 11 | 7 | 1.1 | 8.1 | 1.0 | 3.9 | 3.43 |
| 12 | 7 | 1.2 | 8.2 | 1.1 | 3.9 | 3.76 |
| 13 | 7 | 1.2 | 8.2 | 1.2 | 3.9 | 4.65 |
| 14 | 8 | 1.4 | 9.4 | 1.3 | 3.9 | 3.00 |
| 15-24 | 19 | 4.7 | 23.7 | 1.0 | 8.2 | 2.50 |
| 25-44 | 23 | 6.7 | 29.7 | 1.0 | 3.2 | 5.00 |
| 45-64 | 27 | 5.1 | 32.1 | 3.0 | 11.0 | 5.00 |
| ≥65 | 27 | 10.6 | 37.6 | 6.0 | 11.4 | 0.00 |

^A^ From Wolff 2021 [6].

^B^ Outpatient resource utilization calculated as the sum of primary and specialist care.

^C^ To be conservative, these values were assumed to 3.9 instead of 9.3, 10.2, and 20.6 for 7, 8, 9 years respectively.

## Denmark model calibration

Fig A. Observed and fitted A) varicella seroprevalence and B) herpes zoster incidence

For this Danish adaptation, the model was calibrated to age-stratified varicella seroprevalence and HZ incidence data sets from Norway [12, 13], as this data was unavailable for Denmark. Maximum likelihood estimates of model parameters governing the transmission of varicella infection and the natural history of HZ were found that best fit the observed epidemiological data [12, 13].

## References

1. Agreement of price reductions and a cap on the prices of hospital-only medicinal products for the period April 1, 2019 – March 31, 2023. Available from: <https://www.lif.dk/wp-content/uploads/2020/10/Price-cap-agreement-on-hospital-medicin-2019-2023.pdf>.

2. Szende A, Janssen B, Cabases J, Eds. Self-Reported Population Health: An International Perspective based on EQ-5D. Dordrecht NL: Springer; 2014.

3. Brisson M, Edmunds WJ. Epidemiology of Varicella-Zoster Virus in England and Wales. Journal of medical virology. 2003;70 Suppl 110.1002/jmv.10313.

4. Pellissier JM, Brisson M, Levin MJ. Evaluation of the cost-effectiveness in the United States of a vaccine to prevent herpes zoster and postherpetic neuralgia in older adults. Vaccine. 2007;25:(49). 10.1016/j.vaccine.2007.09.066.

5. Oster G, Harding G, Dukes E, Edelsberg J, Cleary PD. Pain, medication use, and health-related quality of life in older persons with postherpetic neuralgia: results from a population-based survey. The journal of pain. 2005;6:(6). 10.1016/j.jpain.2005.01.359.

6. Wolff E, Widgren K, Scalia Tomba G, Roth A, Lep T, Andersson S. Cost-effectiveness of varicella and herpes zoster vaccination in Sweden: An economic evaluation using a dynamic transmission model. PLoS One. 2021;16:(5). 10.1371/journal.pone.0251644.

7. Praktiserende Lægers Organisation. HONORARTABEL 2020. Available from: <https://www.laeger.dk/sites/default/files/honorartabel_01.10.20.pdf>.

8. World Health Organization. WHO-CHOICE estimates of cost for health service delivery. Available from: <https://www.who.int/choice/country/dnk/cost/en/>.

9. Klein NP, Bartlett J, Fireman B, Marks MA, Hansen J, Lewis E, et al. Long-term effectiveness of zoster vaccine live for postherpetic neuralgia prevention. Vaccine. 2019;37:(36). 10.1016/j.vaccine.2019.07.004.

10. Haugnes H, Flem E, Wisløff T. Healthcare costs associated with varicella and herpes zoster in Norway. Vaccine. 2019;37:(29). 10.1016/j.vaccine.2019.05.063.

11. Heininger U, Pillsbury M, Samant S, Lienert F, Guggisberg P, Gani R, et al. Health Impact and Cost-effectiveness Assessment for the Introduction of Universal Varicella Vaccination in Switzerland. The Pediatric infectious disease journal. 2021;40:(6). 10.1097/inf.0000000000003136.

12. Bollaerts K, Riera-Montes M, Heininger U, Hens N, Souverain A, Verstraeten T, et al. A systematic review of varicella seroprevalence in European countries before universal childhood immunization: deriving incidence from seroprevalence data. Epidemiology and infection. 2017;145:(13). 10.1017/s0950268817001546.

13. Marangi L, Mirinaviciute G, Flem E, Scalia Tomba G, Guzzetta G, Freiesleben de Blasio B, et al. The natural history of varicella zoster virus infection in Norway: Further insights on exogenous boosting and progressive immunity to herpes zoster. PLoS One. 2017;12:(5). 10.1371/journal.pone.0176845.
