# Supplementary material for: Universal varicella vaccination in Denmark: Modeling public health impact, age-shift, and cost-effectiveness
Source: PLOS Glob Public Health. 2023 Apr 5;3(4):e0001743. doi: 10.1371/journal.pgph.0001743 (PMC10075481; doi:10.1371/journal.pgph.0001743)
Supplement: S3 Text — (DOCX) [file pgph.0001743.s003.docx]

# S3 Text: Model results

- [**Main results**](#_Main_results)
- Fig A. Age distribution of annual varicella cases
- Table A. Age distribution of annual varicella cases A
- Fig B. Herpes zoster incidence by time, by vaccination strategy
- Table B. Vaccination costs, treatment costs, and total costs by vaccination strategy A
- [**Sensitivity analyses: Deterministic sensitivity analysis and probabilistic sensitivity analysis**](#_Sensitivity_analyses:_Deterministic)
- Table C. Strategy parameters and values used in the probabilistic and one-way sensitivity analyses
- Fig C. One-way sensitivity analysis on ICER for vaccination strategies from the payer perspective
- Fig D. One-way sensitivity analysis on ICER for vaccination strategies from the societal perspective
- Fig E. Probabilistic sensitivity analysis of incremental cost and incremental QALYs for vaccination strategy versus no vaccination, from the payer perspective
- Fig F. Probabilistic sensitivity analysis of incremental cost and incremental QALYs for vaccination strategy versus no vaccination, from the societal perspective
- **[Scenario analysis](#_Scenario_analysis)**
- Table D. Scenario analyses for each vaccination strategy versus no vaccination from the payer and societal perspectives- Results for no exogenous boosting scenario
- Table E. Scenario analyses for each vaccination strategy versus no vaccination from the payer and societal perspectives- Results for static discounting rate scenarios
- Table F. Scenario analyses for each vaccination strategy versus no vaccination from the payer and societal perspectives- Results for time horizon scenarios

## Main results

Fig A. Age distribution of annual varicella cases

UVV, universal varicella vaccination.

Shown is the age-specific breakdown of varicella cases for no vaccination. Inset shows the age-specific breakdown of varicella cases for strategy C/E.

**Strategy C**: V-MSD (15 months) + V-MSD (48 months); **Strategy E**: V-MSD (15 months) + MMRV-MSD (48 months).

Table A. Age distribution of annual varicella cases ^A^

| **Strategy/**  **Years since UVV** | **Total annual varicella cases, n (%)** | | | | | | |
| --- | --- | --- | --- | --- | --- | --- | --- |
| **No vaccination** | **<1** | **1 to <5** | **5 to <10** | **10 to <15** | **≥15** | **Total** | **>10** |
| 0 | 4,955 (7%) | 26,953 (40%) | 27,271 (40%) | 6,755 (10%) | 2,157 (3%) | 68,091 (100%) | 8,912 (13%) |
| **Strategy A** |  |  |  |  |  |  |  |
| 5 | 336 (11%) | 311 (10%) | 1,184 (37%) | 710 (22%) | 649 (20%) | 3,190 | 1,359 (43%) |
| 10 | 308 (8%) | 225 (6%) | 562 (15%) | 1,469 (38%) | 1,271 (33%) | 3,835 | 2,740 (71%) |
| 15 | 284 (9%) | 206 (6%) | 351 (11%) | 683 (21%) | 1,685 (53%) | 3,209 | 2,368 (74%) |
| 20 | 258 (13%) | 186 (10%) | 302 (16%) | 391 (20%) | 806 (41%) | 1,943 | 1,197 (62%) |
| 50 | 142 (15%) | 104 (11%) | 174 (19%) | 218 (23%) | 302 (32%) | 940 | 520 (55%) |
| **Strategy B** |  |  |  |  |  |  |  |
| 5 | 369 (9%) | 498 (12%) | 1,671 (40%) | 919 (22%) | 711 (17%) | 4,168 | 1,630 (39%) |
| 10 | 349 (6%) | 402 (7%) | 1,031 (18%) | 2,306 (40%) | 1,730 (30%) | 5,818 | 4,036 (69%) |
| 15 | 318 (6%) | 364 (7%) | 697 (13%) | 1,273 (25%) | 2,536 (49%) | 5,188 | 3,809 (73%) |
| 20 | 284 (8%) | 324 (9%) | 595 (17%) | 798 (23%) | 1,449 (42%) | 3,450 | 2,247 (65%) |
| 50 | 156 (8%) | 181 (10%) | 345 (19%) | 470 (26%) | 689 (37%) | 1,841 | 1,159 (63%) |
| **Strategy C** |  |  |  |  |  |  |  |
| 5 | 334 (11%) | 488 (16%) | 1,006 (32%) | 678 (22%) | 641 (20%) | 3,147 | 1,319 (42%) |
| 10 | 309 (9%) | 411 (11%) | 491 (14%) | 1,232 (34%) | 1,168 (32%) | 3,611 | 2,400 (66%) |
| 15 | 289 (9%) | 382 (12%) | 332 (11%) | 610 (20%) | 1,462 (48%) | 3,075 | 2,072 (67%) |
| 20 | 266 (13%) | 350 (17%) | 294 (14%) | 382 (19%) | 752 (37%) | 2,044 | 1,134 (55%) |
| 50 | 146 (14%) | 195 (19%) | 169 (16%) | 217 (21%) | 302 (29%) | 1,029 | 519 (50%) |
| **Strategy D** |  |  |  |  |  |  |  |
| 5 | 379 (9%) | 931 (22%) | 1,323 (32%) | 856 (20%) | 697 (17%) | 4,186 | 1,553 (37%) |
| 10 | 362 (7%) | 862 (16%) | 868 (16%) | 1,784 (33%) | 1,517 (28%) | 5,393 | 3,301 (61%) |
| 15 | 338 (7%) | 803 (16%) | 659 (13%) | 1,107 (22%) | 2,118 (42%) | 5,025 | 3,225 (64%) |
| 20 | 309 (8%) | 731 (20%) | 583 (16%) | 780 (21%) | 1,330 (36%) | 3,733 | 2,110 (57%) |
| 50 | 169 (8%) | 406 (20%) | 337 (16%) | 466 (23%) | 683 (33%) | 2,061 | 1,149 (56%) |

^A^ Values presented as total number of annual cases (% of total annual cases). Years are post-UVV.

Health outcomes with strategies E and F (not shown) were the same as for strategies C and D, respectively.

Fig B. Herpes zoster incidence by time, by vaccination strategy

Shown is HZ incidence by time, by vaccination strategy, for total HZ (including natural and vaccine-type HZ) after the start of universal varicella vaccination. HZ incidences with strategies E and F, which are not shown, were the same as for strategies C and D, respectively.

Table B. Vaccination costs, treatment costs, and total costs by vaccination strategy ^A^

|  | **No vaccination** | **Strategy A** | **Strategy B** | **Strategy C** | **Strategy D** | **Strategy E** | **Strategy F** |
| --- | --- | --- | --- | --- | --- | --- | --- |
| **Vaccination costs** | - | 154,069,725 | 158,666,314 | 147,715,911 | 151,818,769 | 146,320,572 | 152,911,392 |
| **Disease treatment costs** | 1,128,148,326 | 1,106,057,348 | 1,106,263,235 | 1,106,007,953 | 1,106,193,188 | 1,106,007,941 | 1,106,193,255 |
| **Total cost (Payer) ^B^** | 1,128,148,326 | 1,260,127,073 | 1,264,929,549 | 1,253,723,864 | 1,258,011,957 | 1,252,328,513 | 1,259,104,647 |
| **Productivity lost + disease treatment cost** | 1,238,055,064 | 1,117,201,777 | 1,119,463,914 | 1,117,253,029 | 1,119,694,328 | 1,117,253,019 | 1,119,694,280 |
| **Total cost (Societal) ^C^** | 1,238,055,064 | 1,271,271,502 | 1,278,130,228 | 1,264,968,940 | 1,271,513,097 | 1,263,573,591 | 1,272,605,672 |

^A^ Costs are in €.

^B^ Total cost from payer perspective includes varicella vaccination cost (vaccine acquisition and administration costs) + disease treatment cost for varicella and zoster cases.

^C^ Total cost from societal perspective includes productivity loss due to days missed from work by the patient or caregiver for varicella and HZ cases + disease treatment cost + vaccination cost (vaccine acquisition and administration costs.

## Sensitivity analyses: Deterministic sensitivity analysis and probabilistic sensitivity analysis

Table C. Strategy parameters and values used in the probabilistic and one-way sensitivity analyses

| **Parameter definition** | **Base value** | **Low value** | **High value** |
| --- | --- | --- | --- |
| Primary coverage | 0.94 | 0.89 | 0.99 |
| Booster coverage | 0.89 | 0.85 | 0.93 |
| V-MSD waning | 0.833 | 0.792 | 0.875 |
| V-GSK waning | 1.111 | 1.056 | 1.167 |
| V-MSD primary take | 0.903 | 0.858 | 0.948 |
| V-GSK primary take | 0.617 | 0.586 | 0.648 |
| V-MSD booster take | 0.690 | 0.656 | 0.725 |
| V-GSK booster take | 0.834 | 0.792 | 0.876 |
| V-MSD cost per dose | 39.25 | 31.40 | 47.10 |
| V-GSK cost per dose | 40.63 | 32.50 | 48.76 |
| Direct treatment costs | 45.49 | 36.392 | 54.588 |
| Indirect treatment costs | 181.873 | 145.501 | 218.252 |

Fig C. One-way sensitivity analysis on ICER for vaccination strategies from the payer perspective

**Strategy A**: V-MSD (12 months) + V-MSD (15 months); **Strategy B**: V-GSK (12 months) + V-GSK (15 months); **Strategy C**: V-MSD (15 months) + V-MSD (48 months); **Strategy D**: V-GSK (15 months) + V-GSK (48 months); **Strategy E**: V-MSD (15 months) + MMRV-MSD (48 months); **Strategy F**: V-GSK (15 months) + MMRV-GSK (48 months).

Fig D. One-way sensitivity analysis on ICER for vaccination strategies from the societal perspective

**Strategy A**: V-MSD (12 months) + V-MSD (15 months); **Strategy B**: V-GSK (12 months) + V-GSK (15 months); **Strategy C**: V-MSD (15 months) + V-MSD (48 months); **Strategy D**: V-GSK (15 months) + V-GSK (48 months); **Strategy E**: V-MSD (15 months) + MMRV-MSD (48 months); **Strategy F**: V-GSK (15 months) + MMRV-GSK (48 months).

Fig E. Probabilistic sensitivity analysis of incremental cost and incremental QALYs for vaccination strategy versus no vaccination, from the payer perspective

Blue points show the incremental costs and QALYs estimated from the randomly generated parameter sets. The orange point represents base case (no vaccination).

**Strategy A**: V-MSD (12 months) + V-MSD (15 months); **Strategy B**: V-GSK (12 months) + V-GSK (15 months); **Strategy C**: V-MSD (15 months) + V-MSD (48 months); **Strategy D**: V-GSK (15 months) + V-GSK (48 months); **Strategy E**: V-MSD (15 months) + MMRV-MSD (48 months); **Strategy F**: V-GSK (15 months) + MMRV-GSK (48 months).

Fig F. Probabilistic sensitivity analysis of incremental cost and incremental QALYs for vaccination strategy versus no vaccination, from the societal perspective

Blue points show the incremental costs and QALYs estimated from the randomly generated parameter sets. The orange point represents base case (no vaccination).

**Strategy A**: V-MSD (12 months) + V-MSD (15 months); **Strategy B**: V-GSK (12 months) + V-GSK (15 months); **Strategy C**: V-MSD (15 months) + V-MSD (48 months); **Strategy D**: V-GSK (15 months) + V-GSK (48 months); **Strategy E**: V-MSD (15 months) + MMRV-MSD (48 months); **Strategy F**: V-GSK (15 months) + MMRV-GSK (48 months).

## Scenario analysis

Table D. Scenario analyses for each vaccination strategy versus no vaccination from the payer and societal perspectives- Results for no exogenous boosting scenario

| **Strategy** | **Payer** | | | | |  | **Societal** | | | | |
| --- | --- | --- | --- | --- | --- | --- | --- | --- | --- | --- | --- |
|  | **Cost (€)** | **QALYs lost** | **Incremental cost (€)** | **QALYs gained** | **ICER**  **(€/QALY)** |  | **Cost (€)** | **QALYs lost** | **Incremental cost (€)** | **QALYs gained** | **ICER**  **(€/QALY)** |
| No vaccination | 1,125,398,929 | 15,563 |  |  |  |  | 1,235,296,624 | 15,563 |  |  |  |
| Strategy A | 1,255,708,087 | 8,706 | 130,309,157 | 6,857 | 19,005 |  | 1,266,788,524 | 8,706 | 31,491,899 | 6,857 | 4,593 |
| Strategy B | 1,260,579,328 | 8,764 | 135,180,398 | 6,798 | 19,884 |  | 1,273,678,669 | 8,764 | 38,382,045 | 6,798 | 5,646 |
| Strategy C | 1,249,298,283 | 8,701 | 123,899,354 | 6,862 | 18,056 |  | 1,260,470,422 | 8,701 | 25,173,798 | 6,862 | 3,669 |
| Strategy D | 1,253,646,936 | 8,755 | 128,248,007 | 6,807 | 18,840 |  | 1,267,024,624 | 8,755 | 31,728,000 | 6,807 | 4,661 |
| Strategy E | 1,247,902,490 | 8,701 | 122,503,561 | 6,862 | 17,852 |  | 1,259,074,676 | 8,701 | 23,778,051 | 6,862 | 3,465 |
| Strategy F | 1,254,740,049 | 8,755 | 129,341,120 | 6,807 | 19,001 |  | 1,268,117,928 | 8,755 | 32,821,304 | 6,807 | 4,822 |

Table reports the effect of the no exogenous boosting scenario over 50 years from the payer and societal perspectives.

Table E. Scenario analyses for each vaccination strategy versus no vaccination from the payer and societal perspectives- Results for static discounting rate scenarios

| **Strategy** | **Payer** | | | | |  | **Societal** | | | | |
| --- | --- | --- | --- | --- | --- | --- | --- | --- | --- | --- | --- |
|  | **Cost (€)** | **QALYs lost** | **Incremental cost (€)** | **QALYs gained** | **ICER**  **(€/QALY)** |  | **Cost (€)** | **QALYs lost** | **Incremental cost (€)** | **QALYs gained** | **ICER**  **(€/QALY)** |
| **5.0% discount rate** | | | | | |  |  | | | | |
| No vaccination | 875,443,246 | 12,110 |  |  |  |  | 960,730,876 | 12,110 |  |  |  |
| Strategy A | 984,145,391 | 6,932 | 108,702,144 | 5,178 | 20,993 |  | 993,336,890 | 6,932 | 32,606,013 | 5,178 | 6,297 |
| Strategy B | 987,976,621 | 6,977 | 112,533,374 | 5,134 | 21,921 |  | 998,737,077 | 6,977 | 38,006,200 | 5,134 | 7,403 |
| Strategy C | 977,997,630 | 6,928 | 102,554,384 | 5,183 | 19,788 |  | 987,253,519 | 6,928 | 26,522,642 | 5,183 | 5,118 |
| Strategy D | 981,400,226 | 6,969 | 105,956,980 | 5,141 | 20,609 |  | 992,367,167 | 6,969 | 31,636,290 | 5,141 | 6,153 |
| Strategy E | 976,947,221 | 6,928 | 101,503,975 | 5,183 | 19,586 |  | 986,203,066 | 6,928 | 25,472,189 | 5,183 | 4,915 |
| Strategy F | 982,222,652 | 6,969 | 106,779,406 | 5,141 | 20,769 |  | 993,189,610 | 6,969 | 32,458,734 | 5,141 | 6,313 |
| **3.0% discount rate** | | | | | |  |  | | | | |
| No vaccination | 1,221,959,025 | 16,904 |  |  |  |  | 1,341,004,922 | 16,904 |  |  |  |
| Strategy A | 1,363,154,139 | 9,498 | 141,195,115 | 7,406 | 19,064 |  | 1,375,035,836 | 9,498 | 34,030,914 | 7,406 | 4,595 |
| Strategy B | 1,368,320,694 | 9,558 | 146,361,669 | 7,346 | 19,925 |  | 1,382,443,838 | 9,558 | 41,438,916 | 7,346 | 5,641 |
| Strategy C | 1,356,661,095 | 9,491 | 134,702,070 | 7,413 | 18,172 |  | 1,368,657,398 | 9,491 | 27,652,476 | 7,413 | 3,730 |
| Strategy D | 1,361,282,232 | 9,549 | 139,323,208 | 7,355 | 18,942 |  | 1,375,741,332 | 9,549 | 34,736,410 | 7,355 | 4,723 |
| Strategy E | 1,355,137,566 | 9,491 | 133,178,542 | 7,413 | 17,967 |  | 1,367,133,855 | 9,491 | 26,128,933 | 7,413 | 3,525 |
| Strategy F | 1,362,475,210 | 9,549 | 140,516,185 | 7,355 | 19,104 |  | 1,376,934,285 | 9,549 | 35,929,363 | 7,355 | 4,885 |

Table reports the effect of a static discount rate (5.0% and 3.0%) over 50 years from the payer and societal perspectives.

Table F. Scenario analyses for each vaccination strategy versus no vaccination from the payer and societal perspectives- Results for time horizons scenarios

| **Strategy** | **Payer** | | | | |  | **Societal** | | | | |
| --- | --- | --- | --- | --- | --- | --- | --- | --- | --- | --- | --- |
|  | **Cost (€)** | **QALYs lost** | **Incremental cost (€)** | **QALYs gained** | **ICER**  **(€/QALY)** |  | **Cost (€)** | **QALYs lost** | **Incremental cost (€)** | **QALYs gained** | **ICER**  **(€/QALY)** |
| **25 years** | | | | | |  |  | | | | |
| No vaccination | 784,648,585 | 10,854 |  |  |  |  | 861,090,877 | 10,854 |  |  |  |
| Strategy A | 888,898,441 | 6,366 | 104,249,856 | 4,488 | 23,226 |  | 897,803,978 | 6,366 | 36,713,101 | 4,488 | 8,179 |
| Strategy B | 892,423,773 | 6,410 | 107,775,188 | 4,445 | 24,247 |  | 902,805,335 | 6,410 | 41,714,458 | 4,445 | 9,385 |
| Strategy C | 882,608,921 | 6,360 | 97,960,336 | 4,494 | 21,796 |  | 891,528,042 | 6,360 | 30,437,165 | 4,494 | 6,772 |
| Strategy D | 885,718,899 | 6,400 | 101,070,313 | 4,454 | 22,691 |  | 896,205,939 | 6,400 | 35,115,062 | 4,454 | 7,884 |
| Strategy E | 881,688,531 | 6,360 | 97,039,946 | 4,494 | 21,591 |  | 890,607,571 | 6,360 | 29,516,694 | 4,494 | 6,567 |
| Strategy F | 886,439,356 | 6,400 | 101,790,771 | 4,454 | 22,853 |  | 896,926,271 | 6,400 | 35,835,394 | 4,454 | 8,045 |
| **100 years** | | | | | |  |  | | | | |
| No vaccination | 1,424,231,248 | 19,702 |  |  |  |  | 1,562,983,069 | 19,702 |  |  |  |
| Strategy A | 1,468,231,917 | 10,108 | 44,000,669 | 9,594 | 4,586 |  | 1,480,297,182 | 10,108 | -82,685,886 | 9,594 | Dominant |
| Strategy B | 1,472,901,793 | 10,164 | 48,670,544 | 9,538 | 5,103 |  | 1,487,312,361 | 10,164 | -75,670,708 | 9,538 | Dominant |
| Strategy C | 1,461,723,274 | 10,102 | 37,492,025 | 9,600 | 3,905 |  | 1,473,925,102 | 10,102 | -89,057,967 | 9,600 | Dominant |
| Strategy D | 1,465,723,238 | 10,153 | 41,491,990 | 9,549 | 4,345 |  | 1,480,509,433 | 10,153 | -82,473,636 | 9,549 | Dominant |
| Strategy E | 1,459,917,420 | 10,102 | 35,686,171 | 9,600 | 3,717 |  | 1,472,119,186 | 10,102 | -90,863,882 | 9,600 | Dominant |
| Strategy F | 1,467,138,421 | 10,153 | 42,907,173 | 9,549 | 4,493 |  | 1,481,924,494 | 10,153 | -81,058,574 | 9,549 | Dominant |

Table reports the effect of the time horizon (25 and 100 years) from the payer and societal perspectives.
